# Positive correlation between structural disorder of the HIV-1 Gag N-terminal segment and progeny virus particle formation

**DOI:** 10.1128/jvi.00887-25

**Published:** 2025-09-16

**Authors:** Takaaki Koma, Osamu Kotani, Keisuke Kamba, Kei Miyakawa, Taiki Morita, Hiromi Nakamura, Masaru Yokoyama, Naoya Doi, Tomoyuki Kondo, Takashi Nagata, Akihide Ryo, Akio Adachi, Akira Ono, Masato Katahira, Masako Nomaguchi, Hironori Sato

**Affiliations:** 1 Department of Microbiology, Graduate School of Medicine, Tokushima University 13109, Tokushima, Tokushima, Japan; 2 Pathogen Genomics Center, National Institute of Infectious Diseases 119813 , Musashimurayama, Tokyo, Japan; 3 Institute of Advanced Energy, Kyoto University 34807, Uji, Kyoto, Japan; 4 Research Center for influenza and Respiratory Viruses, National Institute of Infectious Diseases 119813 , Musashimurayama, Tokyo, Japan; 5 AIDS Research Center, National Institute of Infectious Diseases 119813 , Musashimurayama, Tokyo, Japan; 6 Department of Virology III, National Institute of Infectious Diseases 119813 , Musashimurayama, Tokyo, Japan; 7 Department of Microbiology and Immunology, University of Michigan Medical School 12266 , Ann Arbor, Michigan, USA; Icahn School of Medicine at Mount Sinai, New York, New York, USA

**Keywords:** Gag, structural disorder, virus particle production, HIV-1

## Abstract

A class of unstructured peptide segments termed disordered regions plays a crucial role in the regulation of protein structure and function. Although the N-terminal region of the matrix (MA) domain of the HIV-1 Gag precursor protein is composed of an unstructured peptide, the mechanisms underlying the regulation of the unstructured state relating to control of viral phenotypes remain unclarified. We examined, in association, the structural, evolutionary, and biological roles of the MA N-terminal region via mutagenesis. Molecular dynamics simulation of a full-length Gag dimer model suggested that an amino acid residue at position 9 in the MA N-terminal region (MA-9) participates in the Gag dimerization. Information entropy analysis indicated that the MA-9 residue is variable in nature, but the hydrophobic amino acid substitution is evolutionarily maladaptive. Disordered region prediction study suggested that single hydrophobic amino acid substitutions at MA-9 reduce the disordered state of the MA N-terminal region. Consistently, NMR analysis indicated that such substitution reduces motional dynamics of the MA N-terminal region and alters the conformation of the MA domain. A site-directed mutagenesis study showed that hydrophobic amino acid substitutions at the MA-9 residue impair, to different degrees, the elementary and overall processes of virus particle formation in the cells. Importantly, the level of the virus particle formation was positively correlated with the level of disorder of the MA N-terminal region. These results indicate that the maintenance of structural disorder and dynamics of the Gag N-terminal segment is regulated by the MA-9 residue and critical for maintaining the optimal production of HIV-1 particles.

**IMPORTANCE:**

A class of unstructured peptide segments termed disordered regions plays crucial roles in the regulation of protein structure and function. Although HIV-1 Gag precursor protein has multiple disordered elements, molecular mechanisms underlying regulation of unstructured state and viral phenotypes largely remain elusive. In this study, by analyzing in association the structural, evolutionary, and virological roles of the disordered N-terminal region of the HIV-1 Gag protein, we show that an amino acid residue at position 9 of the Gag is able to modulate the N-terminal disordered state, and the level of disorder of the Gag N-terminal region is positively correlated with the level of virus particle formation. Our findings gain new insights into molecular mechanisms of regulation of Gag structure and highlight the importance of a previously unappreciated survival strategy of HIV-1—namely, preservation of the Gag N-terminal disorder.

## INTRODUCTION

A class of unstructured peptide segments named disordered regions plays a crucial role in the structural dynamics and function of proteins to yield a wide diversity of proteins with high plasticity. The disordered segment lacks a defined three-dimensional structure, adopts versatile conformations under physiological conditions, and yet plays roles in protein functions, which have given rise to a new disorder—function paradigm in protein science (1–7) as a counterpart to the classical structure–function paradigm. Studies using genome sequence databases indicate that over 40% of the proteins in any eukaryotic proteome include disordered regions (8–10). The abundance of these unstructured components in proteins would enlarge the conformational landscape of proteins and contribute to the plasticity in protein interaction networks and the fitness of organisms. Although the disordered segments are implicated in some human diseases (11), it remains largely elusive how these segments regulate the fitness of an organism in nature.

Viral proteins are often rich in intrinsically disordered regions. Indeed, in some viruses, disordered residues account for as much as 77.3% of individual viral proteins (12). Accumulating evidence indicates that the disordered regions play a critical role in viral replication in the cells by inducing liquid–liquid phase separation for the effective protein interactions (13). The disordered segments of viral proteins may also play a role in the conformational diversity of the viral proteins. Such a role would be particularly relevant to the highly mutable RNA viruses that exhibit exceptionally quick replication and high adaptability using only small amounts of genetic information.

The Gag precursor protein of HIV-1, the major epidemic variant type of HIV, is a multifunctional structural protein that plays a central role in the late stages of viral replication in infected cells (14). Gag is composed of four domains: matrix (MA), capsid (CA), nucleocapsid (NC), and p6. Each domain of Gag is connected with the disordered linkers and has multiple functions to generate infectious viral particles (14). Unstructured segments also exist within individual Gag domains, such as MA, CA, and NC, as well as at the N and C termini of Gag. A disordered segment in CA functions to allosterically regulate the CA structure for effective assembly of Gag and production of infectious virus particles (15, 16). Thus, like many viral proteins, the HIV-1 Gag protein is rich in the disordered regions that are thought to play a regulatory role for viral replication fitness in cells.

The HIV-1 Gag MA domain functions both in the cytoplasm and plasma membrane for the successful production of infectious viral particles (14). The first critical step involving the MA domain is the specific targeting of the newly translated Gag precursor to the plasma membrane. This process is achieved by the interaction between the Gag MA domain and cellular tRNA; tRNA temporally binds to the highly basic region (HBR) near the N-terminus of MA in the cells and is thought to prevent mis-trapping of the Gag precursor in the intracellular vesicle membranes (17–20). The second critical step involving the MA domain is the formation of an assembly platform to promote Gag-mediated interactions of viral components for producing infectious virus particles in the plasma membrane. This step starts with the anchoring of the Gag precursor in the plasma membrane via a myristic acid moiety covalently attached to the N-terminal glycine residue (21–24) and HBR (25–29) of the MA domain. The Gag anchoring to the plasma membrane accelerates Gag–Gag interactions, leading to the formation of the Gag lattice and subsequent membrane curvature (14). The N-terminus of the MA domain again plays a role in inter-Gag interactions and conformational shift during maturation of nascent virus particles (30). In addition, amino acid residues near the MA N-terminus also participate in the effective incorporation of HIV-1 envelope glycoproteins (Env) into the virions (21, 26, 31). Thus, the N-terminus of MA and its neighboring region fulfill multiple functions at distinct steps during the generation of infectious virus particles in the cells. However, how such multiple functions are regulated remains elusive.

In this study, we revisited the role of the N-terminal segment of HIV-1 Gag protein from the viewpoint of the disorder–function paradigm. During our structural and sequence studies on the Gag protein, we noticed the structural, functional, and evolutionary importance of the amino acid residue at position 9 of the N-terminal region of the Gag protein, termed MA-9. We explored the impact of single substitutions at the MA-9 residue on the MA structure and progeny virus particle formation. The obtained data illustrate a hitherto unappreciated mechanism adopted by HIV-1 to optimize progeny virus production in the cells—namely, preservation of the Gag N-terminal disorder.

## RESULTS

### Molecular modeling and structural characterization of the full-length Gag precursor protein of HIV-1

**Fig 1:**
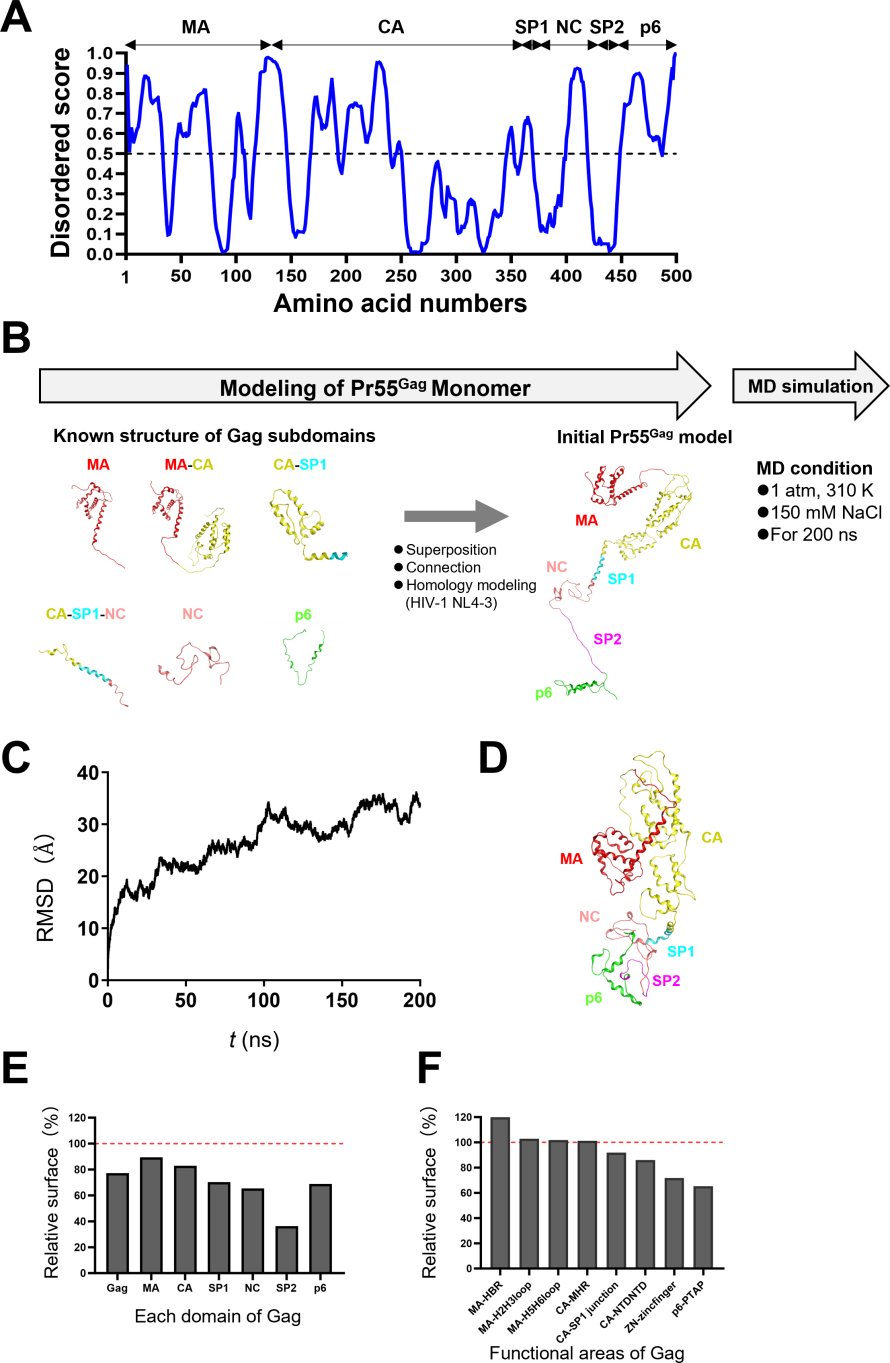
MD-based structural modeling of the full-length Gag precursor of HIV-1. (**A**) Disorder scores of the full-length Gag precursor (Pr55^Gag^) were estimated with PONDR VL-XT predictor (33–35) using the sequence from the HIV-1 NL4-3 strain (GenBank accession no. AF324493) (36). The dashed lines at the Y axis of the figures are the threshold lines for disordered/structured residues. (**B**) A three-dimensional model of the full-length Gag precursor of HIV-1 was constructed by superpositioning reported Gag domain structures, followed by homology modeling using the Molecular Operating Environment (MOE; Chemical Group Inc., Montreal, QC, Canada). The obtained initial Gag precursor model was subjected to MD simulations using modules in the Amber 16 program package (32). (**C**) RMSD between the structure of the initial Gag precursor structure and those at given time points of the MD simulation was calculated using the *cpptraj* module in AmberTools 16 as described previously (37, 38). (**D**) Side view of the Gag precursor model at 200 ns of MD simulation. (**E and F**) Analysis of relative areas of exposed surfaces of Gag domains (**E**) and interaction sites (**F**).

The HIV-1 Gag precursor protein is a multifunctional structural protein that interacts with viral and cellular factors to produce infectious virus particles. The Gag protein has multiple unstructured peptide segments with high disorder scores throughout its structure (Fig. 1A; see Fig. S1 for the disorder scores of the N-terminal end, a region focused in this study), and thereby the overall structure of the full-length Gag protein remains unavailable to date. To gain insights into the overall conformation and the motional dynamics of the Gag protein in solution, we constructed a full-length Gag monomer of the HIV-1 NL4-3 infectious molecular clone as described in Materials and Methods and then subjected this model to molecular dynamics (MD) simulations (Fig. 1B). The root-mean-square deviation (RMSD) (32) between the initial model structure and the structures at given time points of the MD simulation increased rapidly at first and then reached a near plateau with relatively extensive structural fluctuations after 100 ns of the MD simulations (Fig. 1C). Notably, the RMSDs of individual subdomains of Gag did not show such extensive fluctuations after 100 ns of the MD simulations (Fig. S2; MA, CA, SP1, NC, spacer peptide 2 [SP2], and p6). We further examined the RMSDs of multi-domains of Gag monomer. Interestingly, RMSDs of MA-CA and MA-CA-SP1 regions reached a plateau within 200 ns of MD simulations, whereas those of MA-CA-SP1-NC and MA-CA-SP1-NC-SP2 regions were continuously increasing (Fig. S2; MA-CA, MA-CA-SP, MA-CA-SP1-NC, and MA-CA-SP1-NC-SP2). These results suggest that the full-length Gag monomer has an intrinsic property to fluctuate in solution, and the Gag C-terminal region containing NC-SP2 region is critical in causing the continuous fluctuation of HIV-1 Gag monomer in solution.

During the MD simulations, the Gag protein had adopted a more compact conformation as compared with the initial model before the simulation; the MA domain bended to reside in close proximity to the CA domain, and the SP1, NC, SP2, and p6 domains were compactly gathered together (Fig. 1D). Consistent with the general principles of the protein folding in aqueous solution (39, 40), the areas of water-accessible surfaces decreased after MD simulations in all of the Gag domains (Fig. 1E). Furthermore, distances of N-terminal-end-to-C-terminal-end were also reduced in the full-length Gag and all of the Gag domains examined, providing quantitative evidence for the compaction of the Gag monomer during folding in aqueous solution (Fig. S3). Moreover, the overall three-dimensional shape of the Gag model at 200 ns of MD simulations showed striking consistency with the one obtained by the small-angle neutron scattering and hydrodynamic studies with a Gag protein variant lacking the p6 domain (41). The hydrodynamic study, including size-exclusion chromatography and quasi-elastic light scattering analysis, indicated that the hydrodynamic radius (*R_h_*) value of Gag protein with W184A and M185A mutations was 38 Å in solution (41). The sedimentation velocity analysis also indicated a similar *R_h_* of 41 Å for the Gag protein in solution (41). These *R_h_* values were consistent with our results: the distance between N- and C-termini of the full-length Gag monomer model at 200 ns of MD simulation (45 Å). Together, these results support the conclusion that HIV-1 Gag monomer folds into compact conformations in aqueous solution.

Notably, the Gag protein was folded during the MD simulations so that the known Gag interaction surfaces for the later steps of HIV-1 particle formation tended to be less exposed (Fig. 1F). For example, the HBR of the MA domain, which interacts with tRNA for the specific targeting of Gag to the plasma membrane (17–20), remained fully exposed after MD simulation. In contrast, interaction surfaces that were expected to function at a later stage of virion production, such as the CA-SP1 and CA NTD for the Gag lattice assembly, the zinc finger domain for the genome RNA packaging, and p6 PTAP for the virion budding, tended to be less exposed after MD simulation. The results imply that, in addition to the requirements of appropriate trans-acting factors around the Gag protein, the initial conformation of the unliganded Gag may play a role in determining the order of interactions. The results shown in Fig. 1C through F were reproducible with 10 independent MD simulations under the same conditions, suggesting an intrinsically flexible conformation of the HIV-1 Gag monomer in aqueous solution. Taken together, these results show that our MD-simulation-based Gag monomer model is reasonable from the viewpoint of protein chemistry and HIV-1 virology.

### Molecular modeling and structural characterization of the HIV-1 Gag dimer

**Fig 2:**
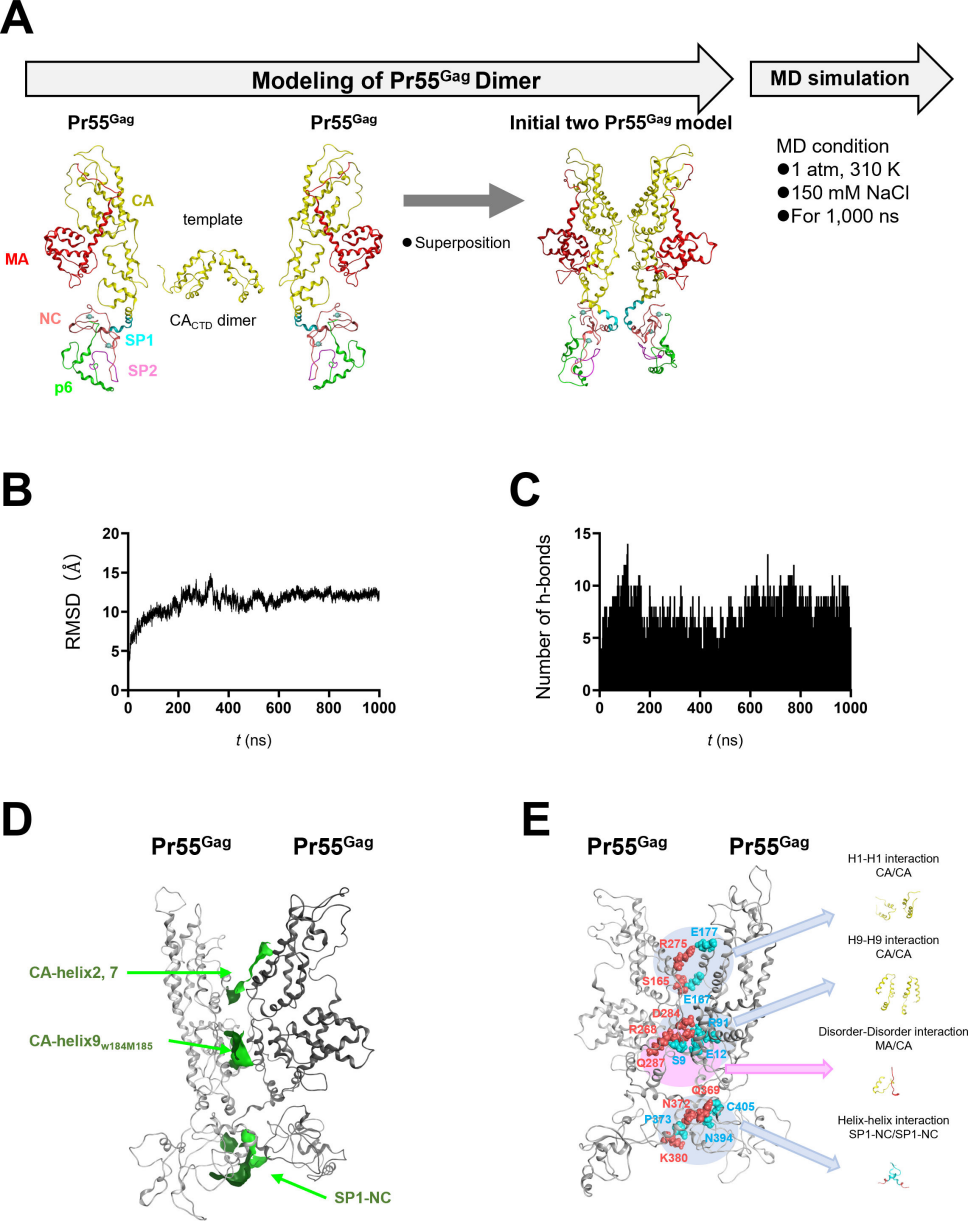
Characterization of molecular interactions between two Gag precursors. (**A**) The Gag precursor model at 200 ns of MD simulation in the equilibrium state under solution conditions was used for modeling of the Gag dimer model using Molecular Operating Environment (MOE). The CA C-terminal domain model (45) was used as a template for the Gag dimer interface. The initial two Gag precursors model was subjected to MD simulation under conditions of 1 atm and 310 K in 150 mM NaCl for 1,000 ns. (**B**) RMSDs between the structure of the initial two Gag precursors model and those at the given time points of the MD simulation are shown. (**C**) Numbers of hydrogen bonds formed between two Gag precursors during 1,000 ns of MD simulations. The trajectory files during 1,000 ns of MD simulations were used to calculate the number of hydrogen bonds between the two Gag precursors using the *cpptraj* module in AmberTools 16 (32). (**D**) Distribution of hydrophobic patches in the binding interface of the two Gag precursors. Hydrophobic patches with a minimum area of 50 Å^2^ for protein–protein interactions were estimated using the Protein Patch Analyzer tool in MOE as described previously (15, 38). (**E**) Visualization of contact sites in the two Gag precursors (red and blue stick models). Panel E depicts the same structure as in panel D but rotated 180°.

HIV-1 Gag–Gag interaction is one of the starting points of the virion formation. To gain insights into the molecular interactions during the Gag dimerization, we constructed a Gag dimer model as described in Materials and Methods and subjected it to MD simulations (Fig. 2A). The RMSDs between the initial dimer structure and the dimers during the MD simulation increased after the onset of the MD simulations and reached a near plateau after 200 ns (Fig. 2B). In contrast to the Gag monomer, the areas of water-accessible surfaces have not decreased during MD simulation: The surface area of the Gag dimer at 1 ns and 1,000 ns was shown to be 54,551.695 Å^2^ and 54,789.055 Å^2^, respectively. These results suggest that significant molecular compaction does not occur during Gag dimerization. Consistent with this data, 5–10 of the hydrogen bonds were rapidly formed between the neighboring monomers and maintained thereafter over 1,000 ns of MD simulations (Fig. 2C). We found three hydrophobic areas that could contribute to dimer formation with hydrophobic interactions along the dimerization interface (Fig. 2D). These three interfaces for the dimerization included the known regions critical for the Gag–Gag interactions, such as the helix–helix interaction between two CA domains (42, 43) and between SP1-NC regions (44). These results suggest that the Gag monomer has an intrinsic conformation that forms a dimer to bury the exposed hydrophobic surfaces of the monomer, once the binding interfaces are arranged in position in close proximity to each other.

Notably, the full-length Gag dimer model disclosed a previously undescribed interaction in addition to the reported ones—i.e., an attractive interaction between the MA and CA domains (Fig. 2E). Serine 9 at the N-terminal disordered region of MA (MA-S9) and glutamine 287 adjacent to the helices 2 and 7 of CA (CA-Q287) had repeatedly formed a hydrogen bond during the MD simulations. The frequency of h-bond formation between MA-S9 and CA-Q287 was 20.19% during 1,000 ns of MD simulation. The hydrogen bond disappeared during the MD simulation when serine 9 was substituted with phenylalanine, suggesting that the MA9 residue plays a role in the adjacency of the two Gag monomers. The hydrogen bond was formed at a site where steric hindrance would not occur by the myristoyl group at glycine 2 of the Gag MA domain (46) (Fig. S4). These results suggest that the MA-9 residue is able to participate in the Gag dimerization. The above results, discussed for Fig. 2B through E, were reproducible with four independent MD simulations under the same conditions, suggesting an intrinsically stable conformation of the HIV-1 Gag dimer in aqueous solution.

### Variation of the amino acid residue at position 9 in the Gag MA domain

**Fig 3:**
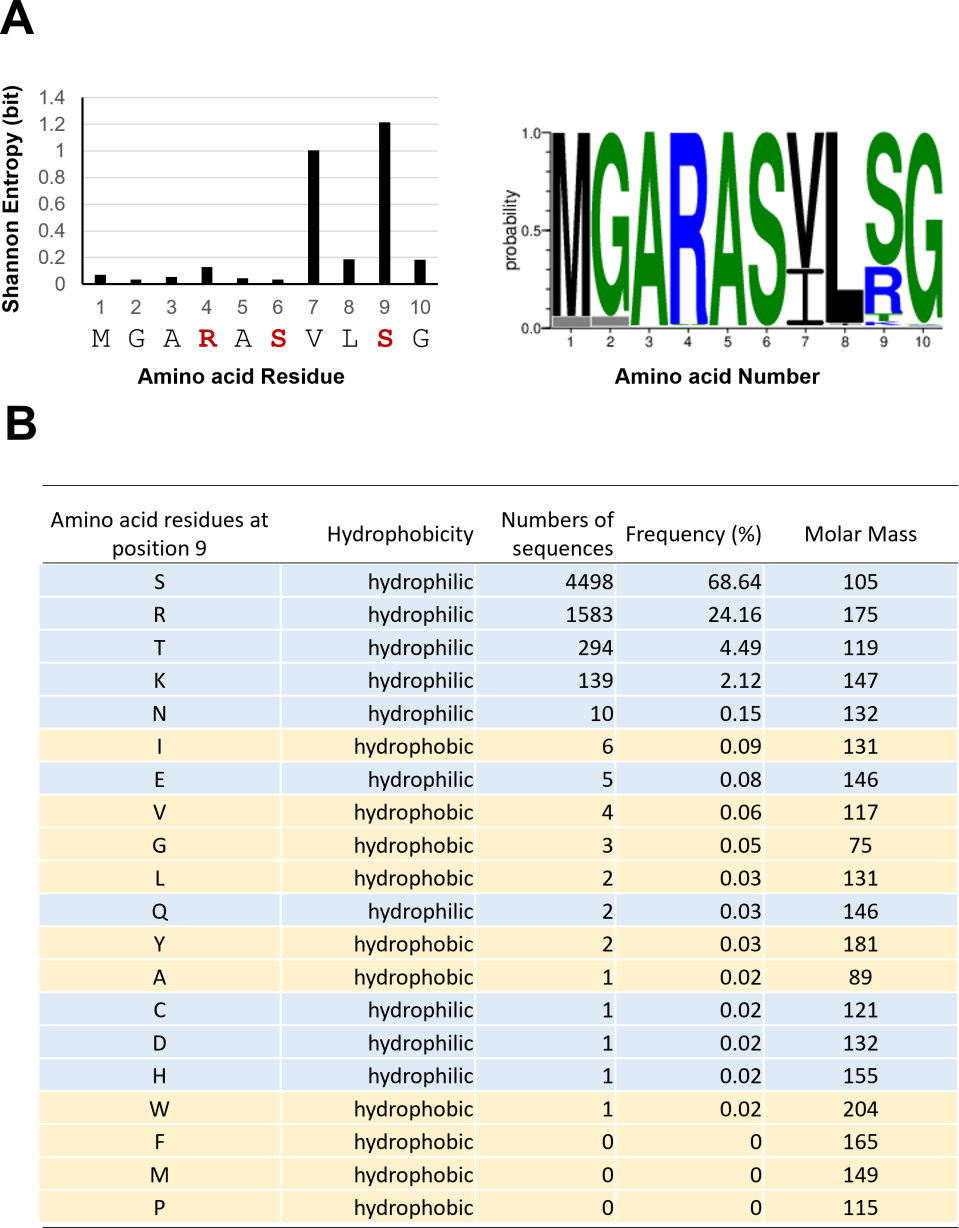
Types and frequencies of amino acid residues present in the N-terminal region of the Gag protein. (**A**) Amino acid variation at positions 1–10 of the N-terminal region of Gag-MA was analyzed with the Shannon entropy method (*n* = 25,222) and AnalyzeAlign (*n* = 6,574). HIV-1 Gag amino acid sequences were obtained from the HIV Sequence Database (http://www.hiv.lanl.gov). Shannon entropy was calculated on the basis of Shannon’s equation (left panel) (49). The red letters indicate hydrophilic amino acids. The AnalyzeAlign tool was used to make the Web Logo of the Gag sequences in February 2022 and to analyze the types and frequencies of amino acid residues at position 9 of Gag after gaps and unspecified amino acid residues were removed (right panel). (**B**) The types, properties, and frequencies of amino acid residues in MA-9 were summarized using the obtained Gag amino acid sequences.

The MA-S9-mediated interaction for the Gag dimerization is unique in that it involved an amino acid in the N-terminal disordered region of MA; thus far, the reported interactions for the Gag dimerization have occurred in residues of the structured helices, i.e., between helix 1 and helix 3 of adjacent MAs, helix 9 and helix 9 of adjacent CA_CTD_s, and helix 12 and helix 12 of adjacent CA-SP1s (30, 42, 44, 47, 48). To clarify the variability of the MA-9 residue *in vivo*, we calculated the Shannon entropy of the individual residues in the MA N-terminus (positions 1–10) using the public HIV sequence database (https://www.hiv.lanl.gov/content/index; Fig. 3A). The amino acid residues of the N-terminal region of Gag-MA were usually highly conserved, but some variations were observed at positions 7 and 9 (Fig. 3A). Among 20 amino acid residues, serine was most abundant at the MA-9 residue, representing about 69% of the Gag sequences (Fig. 3B). Arginine was the next most abundant, representing about 24%. Threonine and lysine were detected as minor populations, representing about a few percentages of the sequences. The number of Gag sequences having other amino acid residues at position 9 was negligible, suggesting induction of severe defects in HIV-1 maintenance in humans. Notably, variants having a hydrophobic amino acid residue at MA-9 were rarely detected in the sequence database, indicating a strong selective disadvantage in human populations. These results indicated that the MA-9 residue is variable, but the hydrophobic amino acid substitution is evolutionarily maladaptive.

### Prediction of effects of single substitutions at MA-9 on the disordered potential of the MA N-terminal region

**Fig 4:**
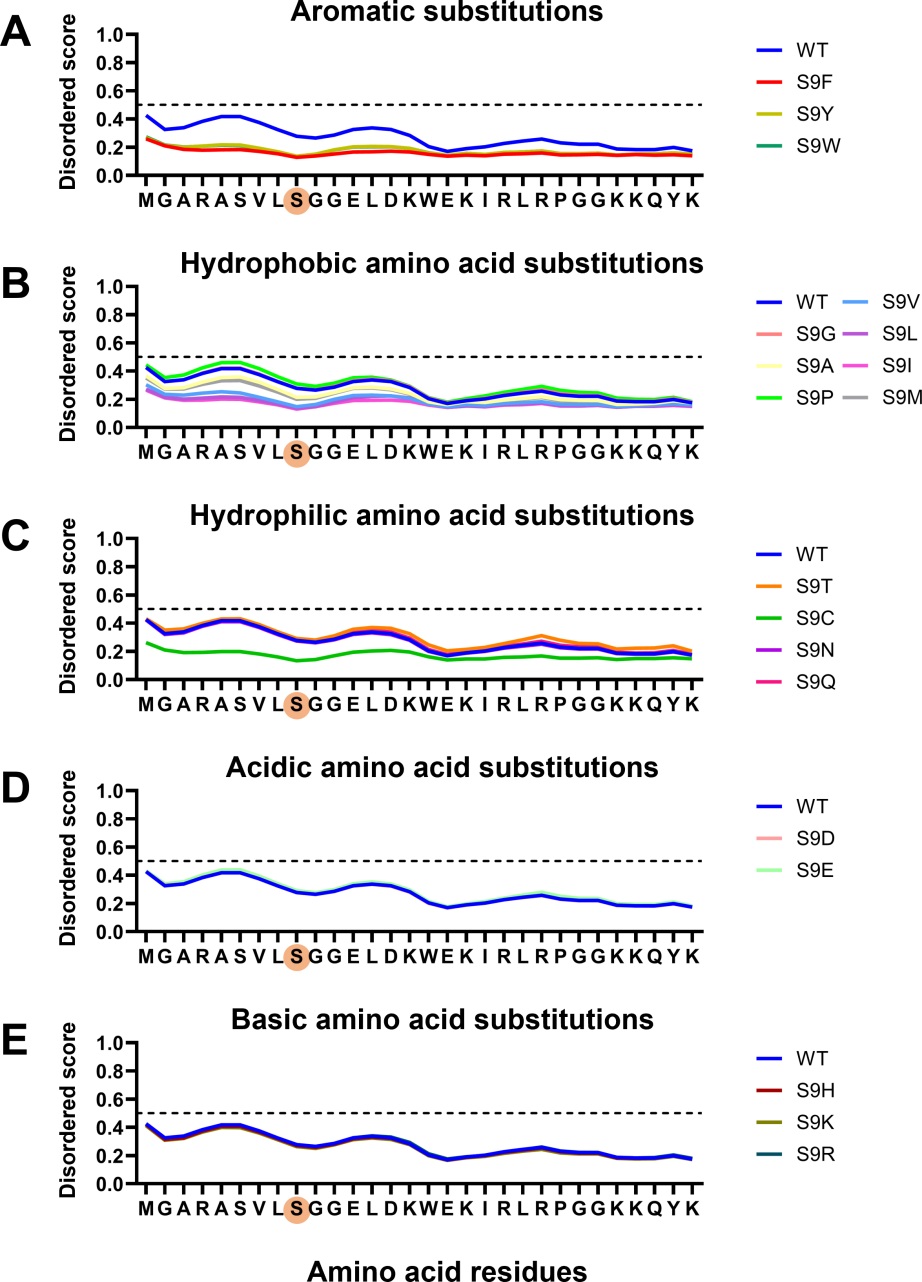
Effects of MA-9 single substitutions on the disordered state of the MA N-terminal region. The disorder level of the N-terminal region of the HIV-1 Gag MA domain was estimated using AIUPred. A score of 0.5 or higher indicates a likely disordered region. Amino acids were classified into five groups according to their properties: aromatic (**A**), hydrophobic (**B**), hydrophilic (**C**), acidic (**D**), and basic (**E**).

Intrinsically disordered segments of proteins generally comprise an insufficient proportion of hydrophobic amino acids to prevent peptide folding (6, 50). Therefore, it is possible that the hydrophobic amino acid substitutions at the MA-9 residue, which were rarely detected in the HIV-1 sequence database, influence the potential of the unstructured state of the MA N-terminal region of the Gag monomer. To address this issue, we examined the effects of a single amino acid substitution at MA-9 on the disordered state of the Gag MA N-terminus (Fig. 4). The disordered levels of individual amino acid residues were estimated with AIUPred (51), a meta-predictor of intrinsically disordered amino acids. The dashed lines at the Y axis of the figures are the threshold lines for disordered/structured residues. The prediction of disorder levels showed that the disorder scores were near 0.5 in the first nine amino acids, higher than the scores in the region downstream of the MA-9 residue (Fig. 4, wild type [WT]). These results are consistent with those of a previous NMR-based analysis, in which the segment containing the first nine amino acids was found to be disordered (46).

Notably, aromatic substitutions at MA-9 (S9F, S9Y, and S9W) all induced drastic reductions in the disorder scores of the MA N-terminal region (Fig. 4A). Similarly, hydrophobic amino acid substitutions often reduced the disordered state of the MA N-terminus (Fig. 4B). In contrast, changes in the disorder scores were mild with hydrophilic amino acid substitutions, including acidic/basic amino acid substitutions (Fig. 4C through E); indeed, with the exception of the substitution to cysteine, these scores were similar to the WT score. The amino acid type-dependent changes in the disorder profiles were reproducible with another predictor of disordered regions, the Predictor of Natural Disordered Protein Regions (PONDR; Fig. S5) (33–35). These data suggest that the N-terminus of the Gag proteins becomes more structured compared to that of the WT when a hydrophobic amino acid substitution occurs at the MA-9 residue.

### Dynamic properties of the N-terminal regions of Gag-MA and Gag-MA-S9F

The structural dynamics of proteins in solution play key roles in molecular interactions (52–54). Accordingly, we next used NMR to examine the potential role of MA-9 in the regulation of dynamic properties of the N-terminal region of the MA domain. For this purpose, we compared the signal pattern in the two-dimensional (2D) ^1^H–^15^N HSQC spectrum of ^15^N-labeled Gag MA 6His between HIV-1 NL4-3 MAs from the WT and those from the MA-S9F mutant, which was entirely absent from the HIV-1 Gag sequence database (Fig. 3B) and exhibited a reduction in the disordered state of the Gag MA N-terminus (Fig. 4A).

**Fig 5:**
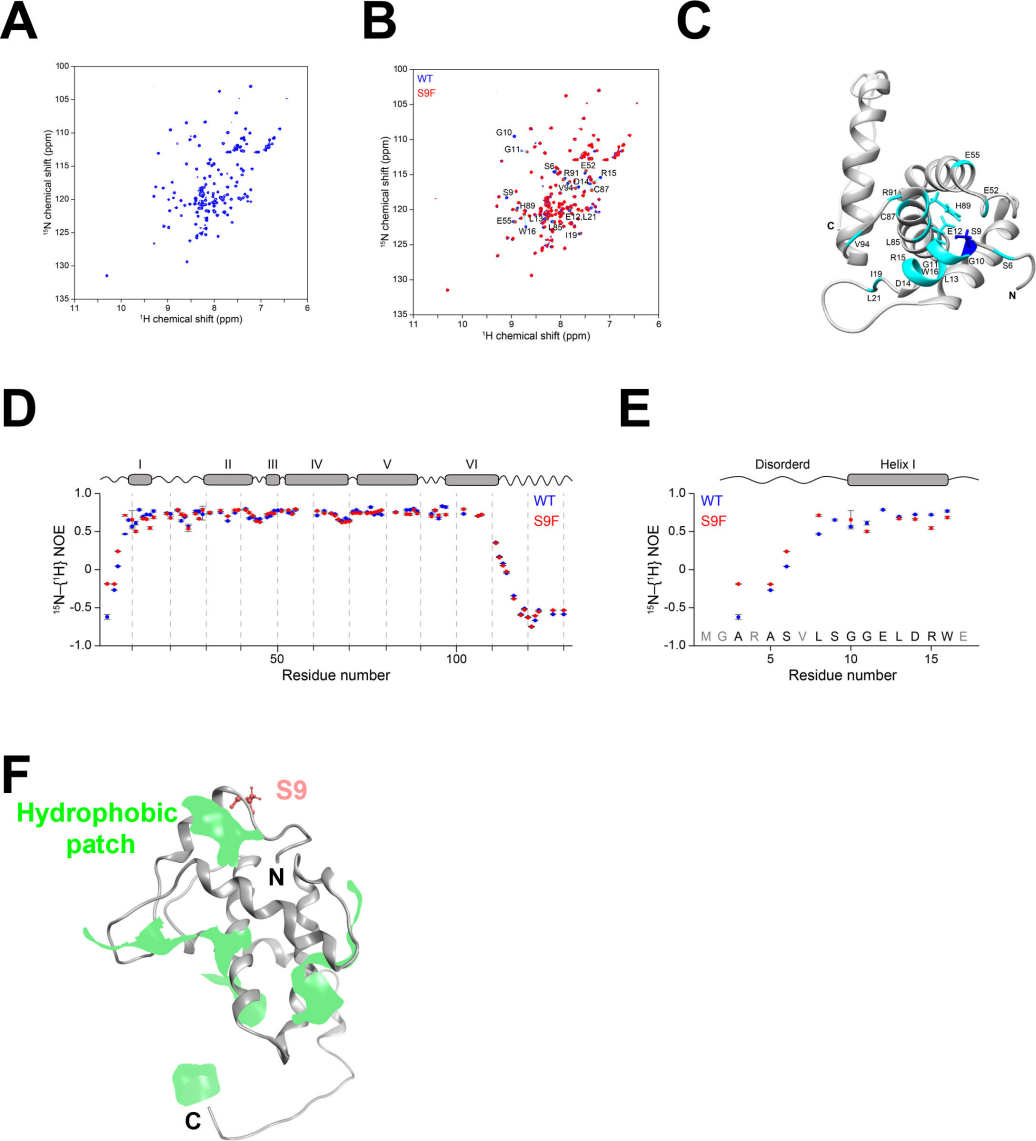
Motion of the Gag-MA N-terminal region. (**A**) 2D ^1^H-^15^N HSQC spectrum of 100 µM ^15^N-labeled Gag-MA 6His at 35°C and pH 5.5. (**B**) Superimposition of the 2D ^1^H-^15^N HSQC spectra of ^15^N-labeled Gag-MA 6His and ^15^N-labeled Gag-MA-S9F 6His at 35°C. Signals exhibiting chemical shift perturbations are labeled with the residue number and a one-letter code. (**C**) Mapping of residues with profound chemical shift perturbation upon the introduction of the S9F substitution on the Gag-MA structure (PDB ID: 2H3F). (**D**) Steady-state NOE values are shown for ^15^N-labeled Gag-MA 6His (blue) and ^15^N-labeled Gag-MA-S9F 6His (red). (**E**) Expansion of the N-terminal region of Gag-MA. The positions of α-helical regions are shown at the top. (**F**) Molecular patches relevant to hydrophobic interactions. Light green portions indicate the hydrophobic patches. The N- and C-termini of Gag-MA are shown by the letters “N” and “C,” respectively.

The signal pattern in the 2D ^1^H–^15^N HSQC spectrum of ^15^N-labeled Gag-MA 6His closely resembled that reported by Massiah et al. (Fig. 5A) (55). The assignment of the 2D ^1^H–^15^N HSQC spectrum of ^15^N-labeled Gag-MA 6His was performed based on Massiah’s report provided at pH 8.0 and a series of 2D ^1^H–^15^N HSQC spectra obtained under different pH conditions. Signal assignment was achieved for 58% of the spectrum.

Then, we compared the 2D ^1^H–^15^N HSQC spectra of ^15^N-labeled Gag-MA 6His and ^15^N-labeled Gag-MA-S9F 6His (Fig. 5B). The signals of many residues did not change upon the S9F mutation, indicating that this mutant has a similar, if not identical, structure to the WT. However, the signals from residues S6, G10, G11, E12, L13, D14, K15, W16, I19, L21, E52, E55, L85, C87, H89, R91, and V94 displayed some chemical shift changes. We mapped these affected residues onto the previously reported structure of Gag MA (PDB ID: 2H3F) (46) (Fig. 5C). Residues G10, G11, E12, L13, D14, K15, W16, I19, and L21 are within helix I (S9–E17). Considering that S9 in the WT forms the N-terminal cap in helix I, the S9F substitution might have perturbed the conformation of helix I. In addition, residues E52, E55, L85, C87, H89, R91, and V94 were found to be in close proximity in three-dimensional space to S9 (Fig. 5C). The earlier structure indicated a hydrogen-bonding network involving S9, E12, and H89 (46). Hence, the S9F mutation might have also affected the conformation of the C-terminal region of helix V, to which H89 belongs.

We deduced backbone ^1^H-^15^N heteronuclear NOEs to inspect the dynamic properties of Gag-MA and Gag-MA-S9F (Fig. 5D and E). The values of ^1^H-^15^N heteronuclear NOE for the residues in α-helical regions of Gag-MA were ~0.8, indicating structural rigidity in these α-helices. The NOE values of Gag-MA in the regions connecting these α-helices ranged between ~0.6 and 0.8, suggesting fast internal motions in these regions. The NOE values of residues in the N- and C-terminal regions were below 0.6, indicating disorder in these regions.

We compared the NOE values of the N-terminal residues A3–L8 between Gag-MA and Gag-MA-S9F. The NOE values of residues A3, A5, S6, and L8 were found to be larger for Gag-MA-S9F than for Gag-MA. This suggests that the internal motion of the disordered N-terminal region was suppressed upon the S9F substitution to some extent. We hypothesize that the motional suppression in the disordered N-terminal region is a result of the bulkiness of F9, which could lead to a hydrophobic interaction involving F9 and hydrophobic residues on the molecular surface (Fig. 5F). Together, these results suggest that the hydrophobic amino acid substitution at MA-9 can reduce motional dynamics of the MA N-terminus and influence the conformation of the MA domain.

### Virion production is drastically reduced by substitutions of Gag-MA-S9 with amino acid residues having aromatic and hydrophobic properties

To investigate the biological role of the MA-9 residue in the HIV-1 life cycle, we conducted site-directed mutagenesis using the HIV-1 NL4-3 proviral clone. Mutations at position 9 in Gag-MA (S9R/P/G/H/A/E/I/L/Y/F/W) were selected based on their frequency in the HIV sequence database and the chemical properties of the amino acids (Fig. 3B). Given that the Gag-MA S9 residue can be important for Gag dimerization, virion production is expected to be influenced by mutations at the site. Two well-known virion production-deficient mutant clones were generated and used as controls. One is a Gag-MA-G2A mutant clone that lacks Gag plasma membrane-targeting activity via a defect in the N-terminal myristoylation site of Gag. The other is a Gag-CA-WM184/185AA (WMAA) mutant clone lacking Gag dimerization ability due to mutations within the Gag-CA C-terminal domain (CTD) (20, 56–58).

**Fig 6:**
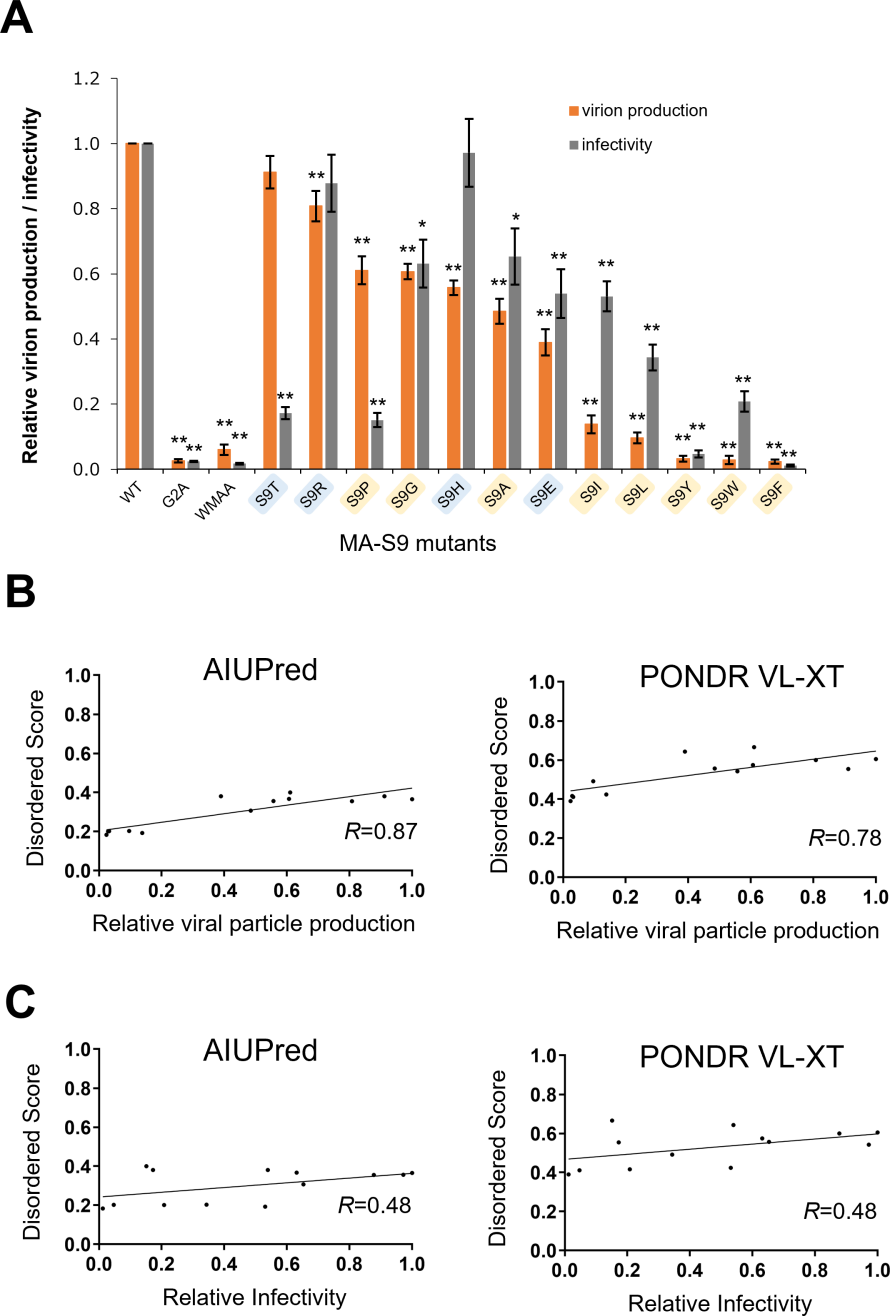
Effects of Gag mutations on virion production and viral infectivity and relation between the disordered state and viral phenotype. (**A**) HEK293T cells were transfected with the indicated full-length proviral clones. The culture supernatants were collected at 24 h post-transfection to measure virion production by reverse transcriptase (RT) assays. RT activity is shown relative to that of NL4-3. Equal amounts of viruses, as determined by RT assays, were inoculated into TZM-bl cells. Cell lysates were prepared on day 2 post-infection for luciferase assays. Infectivity is presented as luciferase activity relative to that of NL4-3. Mean values ± SE from at least four independent experiments are shown. Significance relative to NL4-3 was determined by the Welch’s *t*-test (**P < 0.01; *P < 0.05). Gag-MA-G2A and Gag-CA-WMAA mutant clones were used as controls. (**B**) The relation between the mean disorder scores of the MA-NTD segment and the values of relative viral particle production. The mean disorder scores of the first nine amino acid residues of the Gag MA N-terminus were obtained for individual MA-9 mutants using the data from Fig. 4 (AIUPred [51]; left panel) and Fig. S5 (PONDR VL-XT [33–35]; right panel). The values were used to assess the relation between the disorder level and viral production in Fig. 6A (orange bars). *R*: Pearson’s correlation coefficient. (**C**) The relation between the mean disorder scores of the MA-NTD segment and relative viral infectivity values. The mean disorder scores were used to assess the relation between the level of disorder and viral infectivity values in Fig. 6A (gray bars).

We first examined the effect of Gag-MA S9 mutations on virion production and viral infectivity. Virion production was monitored by measuring Gag-p24 levels in the supernatants from HEK293T cells transfected with proviral clones. Viral infectivity was assessed by measuring the luciferase activity in TZM-bl cells infected with various mutant viruses. As expected from previous reports (14, 59), the control mutants (Gag-MA-G2A and Gag-CA-WMAA) displayed a drastic reduction in both virion production and viral infectivity as compared with the WT NL4-3 (Fig. 6A).

Notably, a majority of the amino acid substitutions at position 9 influenced the ability of the virus to produce virus particles and/or infectious virus particles in a manner that was dependent on the type of amino acid residue substituted (Fig. 6A). Among the S9 mutants tested, the effects of aromatic substitutions at position 9 (Gag-MA-S9Y/F/W) were prominent and were associated with a marked reduction both in virion production and viral infectivity to levels comparable to those of the control mutants (Gag-MA-G2A and Gag-CA-WMAA). In contrast, the effects of S9R substitution were relatively mild, with the levels of both virion production and viral infectivity of the Gag-MA-S9R mutant being comparable with those of the WT. Interestingly, S9T and S9P mutants retained a capacity for virion production similar to that of the WT, yet the viral infectivity was markedly reduced to around 20% that in NL4-3. These results are generally consistent with the differential frequencies of these mutants observed in the field (Fig. 3B). Collectively, they indicate that the MA-9 residue plays key roles in the viral life cycle and survival.

### Identification of the positive correlation between the disorder level of the HIV-1 Gag MA domain N-terminus and the level of HIV-1 particle production

We next examined the relation between the levels of the virus particle production and the level of disorder of the Gag MA N-terminus. To assess this issue, the mean disorder score of the first nine amino acid residues of the Gag MA N-terminus, which form the N-terminal unstructured cap upstream of helix I (Fig. 5C), was calculated for individual MA-9 mutants, and the relation between this score and the relative viral particle production was examined. We found that these structural and biological values correlated positively with good reproducibility when the disorder scores were calculated with two distinct predictors of the disordered state (Fig. 6B, left and right panels; Pearson’s correlation coefficient *R* = 0.87 and 0.78 for AIUPred [51] and PONDR VL-XT [33–35], respectively). The positive correlation was also reproducible when the mean disorder scores were calculated with the first 20 and 30 amino acid residues (*R* = 0.88 and 0.78 for AIUPred and PONDR VL-XT, respectively). These results suggest that the disordered state of the Gag N-terminus plays a key role in the optimal production of HIV-1 particles.

Such a positive correlation was less evident when the viral infectivity was used for the counterpart of the correlation analysis (Fig. 6C; Pearson’s correlation coefficient *R* = 0.48 for both the AIUPred- and PONDR VL-XT-mediated calculations of the disorder scores). We found no correlation between the detection frequency of a particular amino acid residue at the MA-9 site in the sequence database (Fig. 3B) and the Gag MA N-terminal disorder (Fig. 4; Pearson correlation coefficient *R* of −0.1865 and −0.0979 for AIUPred and PONDR VL-XT prediction, respectively). These results suggest that additional factor(s) are involved and should be considered when establishing the fitness of HIV-1 *in vivo*.

### Gag-MA-S9T/P mutations prevent the Env incorporation into virions

**Fig 7:**
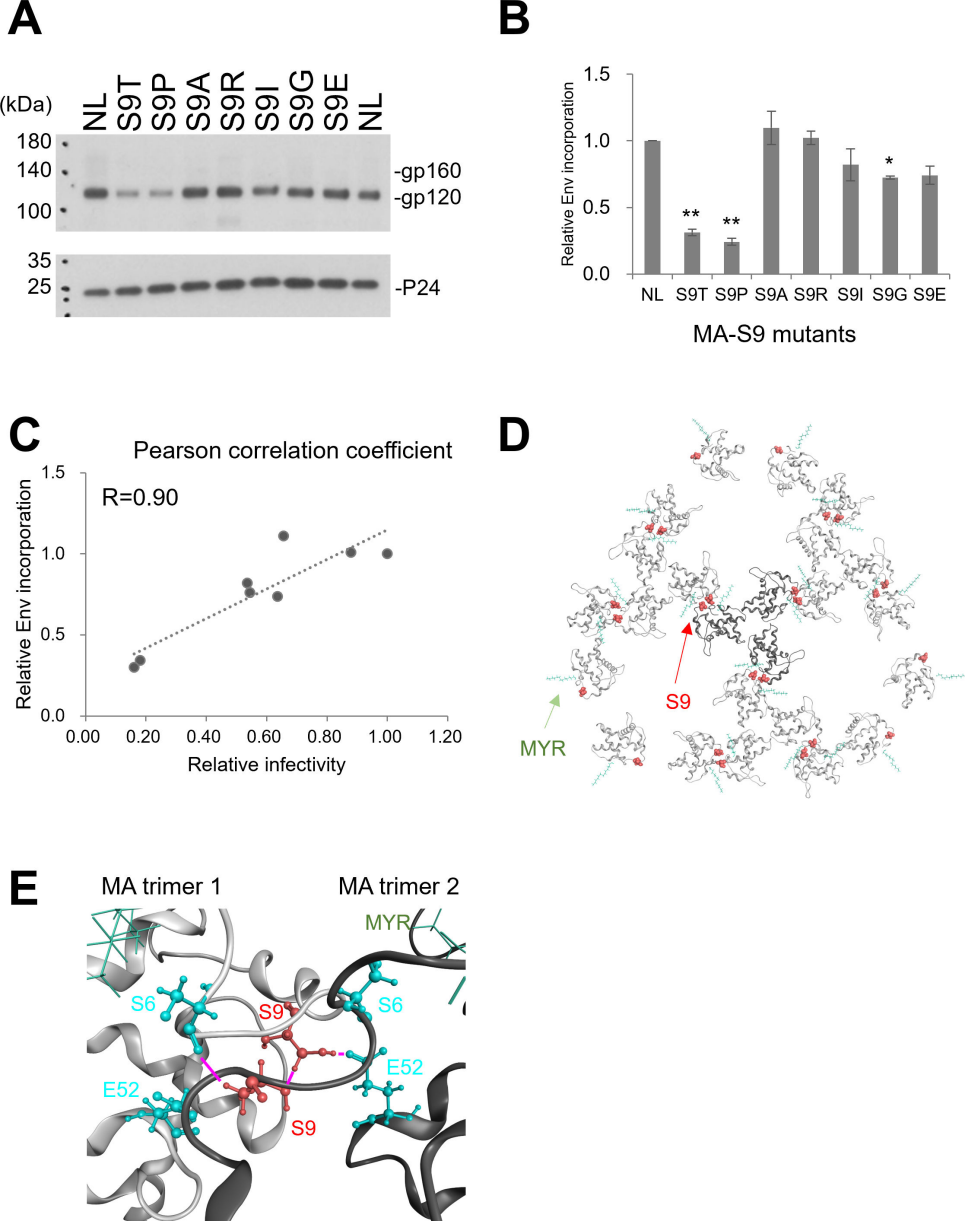
Effect of Gag-MA-S9 mutations on Env incorporation into virions. (**A**) The supernatants from HEK293T cells transfected with the indicated proviral clones were ultracentrifuged through a sucrose cushion, and the pellet samples were harvested for monitoring Env and Gag-p24 in virions. Equal amounts of samples (10 ng p24 for Env and 0.5 ng p24 for Gag) were analyzed by the Western blotting method. (**B**) Representative immunoblotting data from three independent experiments (performed twice for both S9R and S9E) are presented. Env incorporation into virions is presented as the normalized signal intensity (Env/p24) of each sample relative to that of NL4-3 (mean ± SE). The statistical analysis was conducted by Welch’s *t*-test. **P < 0.01; *P < 0.05. (**C**) The correlation between the relative Env incorporation and relative infectivity is indicated by the *R*-score. (**D**) Three-dimensional locations of the MA-S9 amino acid residue in the immature HIV-1 MA model. The immature HIV-1 MA trimer structure is derived from PDB code 7OVQ (30). (**E**) Molecular interactions between two MA trimers. The magenta-colored bars indicate hydrogen bonds.

We next examined the molecular mechanisms by which single substitutions at the MA-9 site impaired the production of infectious virus particles. The S9T and S9P mutants exhibited greater than 60% of the progeny virion production of the WT. Notably, however, the infectivities of the produced virions were markedly impaired to less than 20% of those in the WT (Fig. 6A). To gain insights into the mechanisms underlying these findings, we examined whether the decrease in infectivity of Gag-MA-S9T/P clones is associated with their Env incorporation into virions (Fig. 7). HEK293T cells were transfected with several proviral clones, and on day 2 post-transfection, virions were collected by ultracentrifugation for analysis by Western blotting using anti-Gag-p24 and anti-Env antibodies. The other Gag-MA-S9 mutant clones that exhibited more than 50% infectivity to NL4-3 (S9R/A/I/G/E; Fig. 4) were selected for comparative analysis.

As shown in Fig. 7A and B, the Env incorporation into virions for Gag-MA-S9T/P was reduced to around 30% of that for NL4-3. Such a marked reduction was not observed with Gag-MA-S9R/A/I/G/E mutants, which retained levels of Env incorporation of more than 70% relative to NL4-3. The Env incorporation into virions in the Gag-MA mutants tested was well correlated with their infectivity (Fig. 7C; Pearson’s correlation coefficient, *R* = 0.90). These results indicate that the Gag-MA-S9T/P mutations reduced viral infectivity likely by impairing Env incorporation into virions.

HIV-1 Env incorporation into virions is assumed to occur during or after Gag lattice formation in the plasma membrane. MA-S9 is located at the interface between two MA trimers in the Gag lattice (Fig. 7D). In our experiments, MA-S9 residues on one MA monomer interacted with serine 6, serine 9, and glutamic acid 52 on the neighboring MA monomer by forming hydrogen bonds (Fig. 7E). This implies that the MA-9 residue can participate in the intermolecular interactions during self-assembly of Gag. Collectively, the above results suggest that MA-S9 may be involved in the regulation of interactions between Gag and Env or other trans-acting factors for the Env incorporation into virions.

### Gag-MA mutations that dramatically decrease virion production impair the Gag oligomerization ability

**Fig 8:**
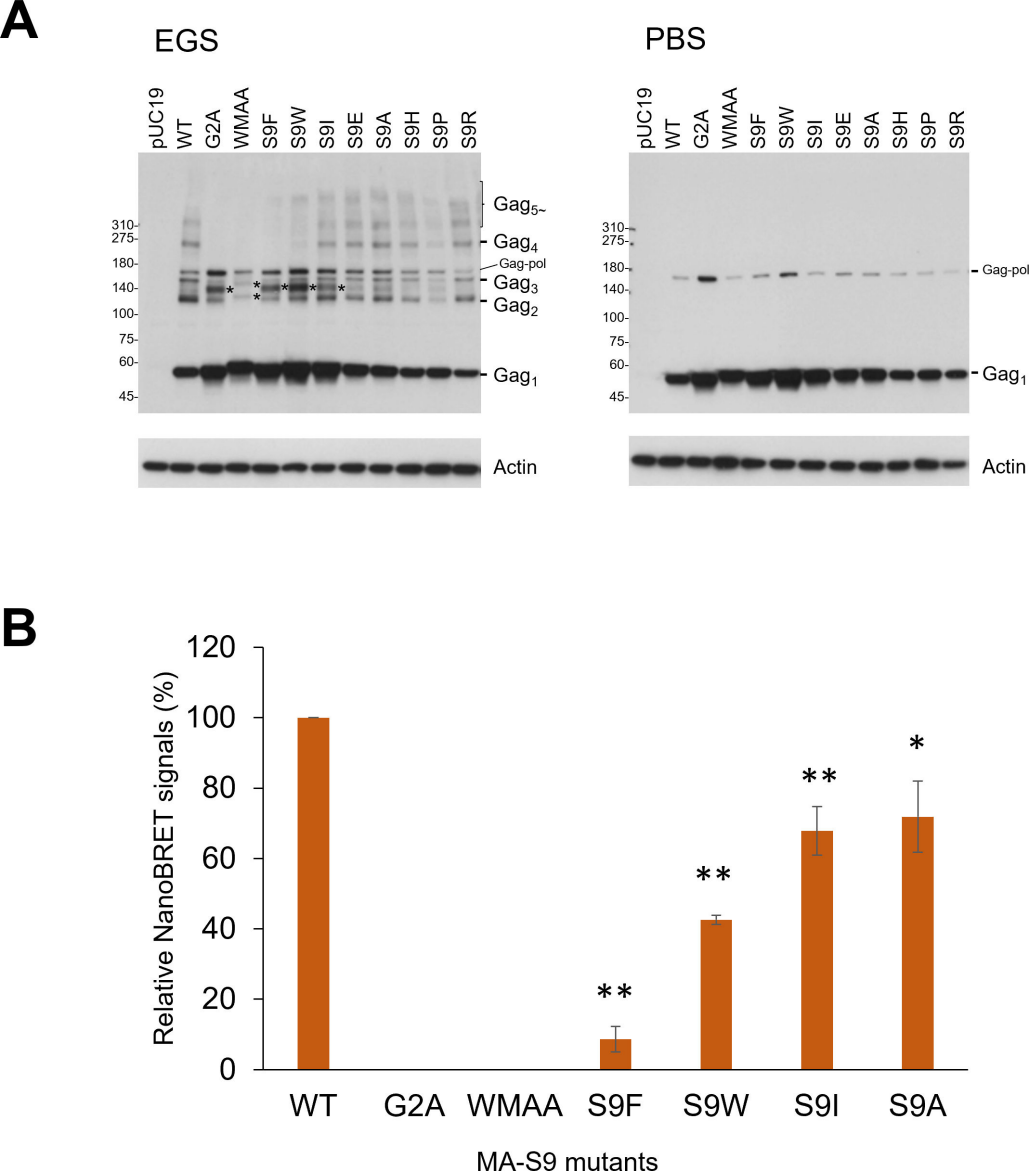
Effect of Gag mutations on Gag oligomerization in cells. (**A**) HeLa cells were transfected with the indicated proviral mutants derived from pNL4-3ΔPro/ΔEnv as a parental WT clone. At 24 h post-transfection, cells were treated with 0.1 mM EGS (left panel) or PBS (right panel) for 30 min. After quenching the reactions, cell lysates were prepared, and equal amounts of protein from the cell lysates (5 µg) were subjected to Western blot analysis using anti-Gag and anti-β-actin antibodies. Plasmid pUC19 and Gag mutants designated Gag-MA-G2A and Gag-CA-WMAA were used as controls. Predicted migration positions of Gag monomer (Gag1), dimer (Gag2), oligomerized Gag (Gag3, Gag4, and Gag5), and the residual Gag-Pol proteins are indicated at right. Representative data from three independent experiments are shown. *Unspecified bands. (**B**) Gag–Gag interactions were detected by NanoBRET. NanoBRET data are shown as values relative to the WT after subtracting the signal value of the dimerization-defective mutant WMAA (*n* = 6; mean ± SE). NanoBRET values for G2A were lower than those for WMAA and are plotted as 0. Significance relative to WT was determined by Welch’s *t*-test. **P < 0.01; *P < 0.05.

Since certain mutations of Gag-MA-S9 drastically reduced the virion production level (Fig. 6A), we next examined the effect of Gag-MA-S9 mutations (R/P/H/A/E/I/W/F) on the Gag oligomerization in cells by the EGS-crosslinking method following a protocol similar to that previously reported (60). HeLa cells were transfected with ΔPro/ΔEnv proviral clones, and then the cells were treated with EGS or PBS. After preparing the cell lysates, we evaluated the Gag-oligomerization state based on the migration position of Gag proteins in the Western blot using an anti-Gag antibody. Gag-MA-G2A and Gag-CA-WMAA ΔPro/ΔEnv clones were used as controls. For PBS treatment without EGS (Fig. 8A, right panel), Gag monomer (Gag_1_) proteins were mainly observed for all clones tested along with small amounts of Gag-Pol proteins. For EGS-crosslinked samples (Fig. 8A, left panel), WT NL4-3ΔPro/ΔEnv produced Gag dimer (Gag_2_), Gag trimer (Gag_3_), Gag tetramer (Gag_4_), and oligomerized Gag (Gag_5~_) products at the predicted migration positions. As expected, for the Gag-CA-WMAA clone, the Gag monomer was predominantly observed, and no oligomerized Gag (Gag_2_~Gag_5~_) was detected. For the Gag-MA-G2A clone, the production of Gag dimer proteins was significantly reduced relative to that for the WT, and no oligomerized Gag products larger than the Gag dimer were observed. Six of the Gag-MA-S9 mutant ΔPro/ΔEnv clones (S9R/P/H/A/E/I) expressed Gag dimer (Gag_2_), Gag trimer (Gag_3_), Gag tetramer (Gag_4_), and oligomerized Gag (Gag_5~_) products in a manner similar to the WT, although the band intensities of these products were different.

Importantly, the GA-MA-S9F/W clones, which have drastically reduced virion production abilities, exhibited a similar phenotype to Gag-MA-G2A. Although a decrease in Gag dimer products was observed for all Gag-MA-S9 mutants tested, Gag oligomerization (Gag_3_~Gag_5~_) of GA-MA-S9F/W clones basically did not proceed beyond Gag dimer formation, which was also the case for Gag-MA-G2A. These results suggested that the inhibition of virion production by MA-G2A and MA-S9F/W mutations results from defects in Gag oligomerization.

This possibility was supported by the results of another assay using a nano-bioluminescence resonance energy transfer (NanoBRET) system to directly measure the efficiency of Gag–Gag interactions in living cells (61, 62) (Fig. 8B). In this assay, MA-S9F/W mutants showed greater reduction in NanoBRET signal production as compared with the other MA-9 mutants, although the impairments were less pronounced than those in the MA-G2A and MA-WMAA mutants. Consistent with the results of the cross-linking experiments in Fig. 8A, the S9I mutant, which exhibited relatively severe defects in progeny virion production, somehow showed only moderate effects on NanoBRET signal production. Consistent with the results of the virion production in Fig. 6A and the cross-linking experiments in Fig. 8A, the effects of the S9A mutation on the NanoBRET signal were much milder than the effects observed in the mutants MA-S9F and MA-S9W with aromatic substitutions at position 9 of MA.

### Virion production-deficient Gag-MA mutants exhibit a drastic decrease in membrane localization of Gag proteins

**Fig 9:**
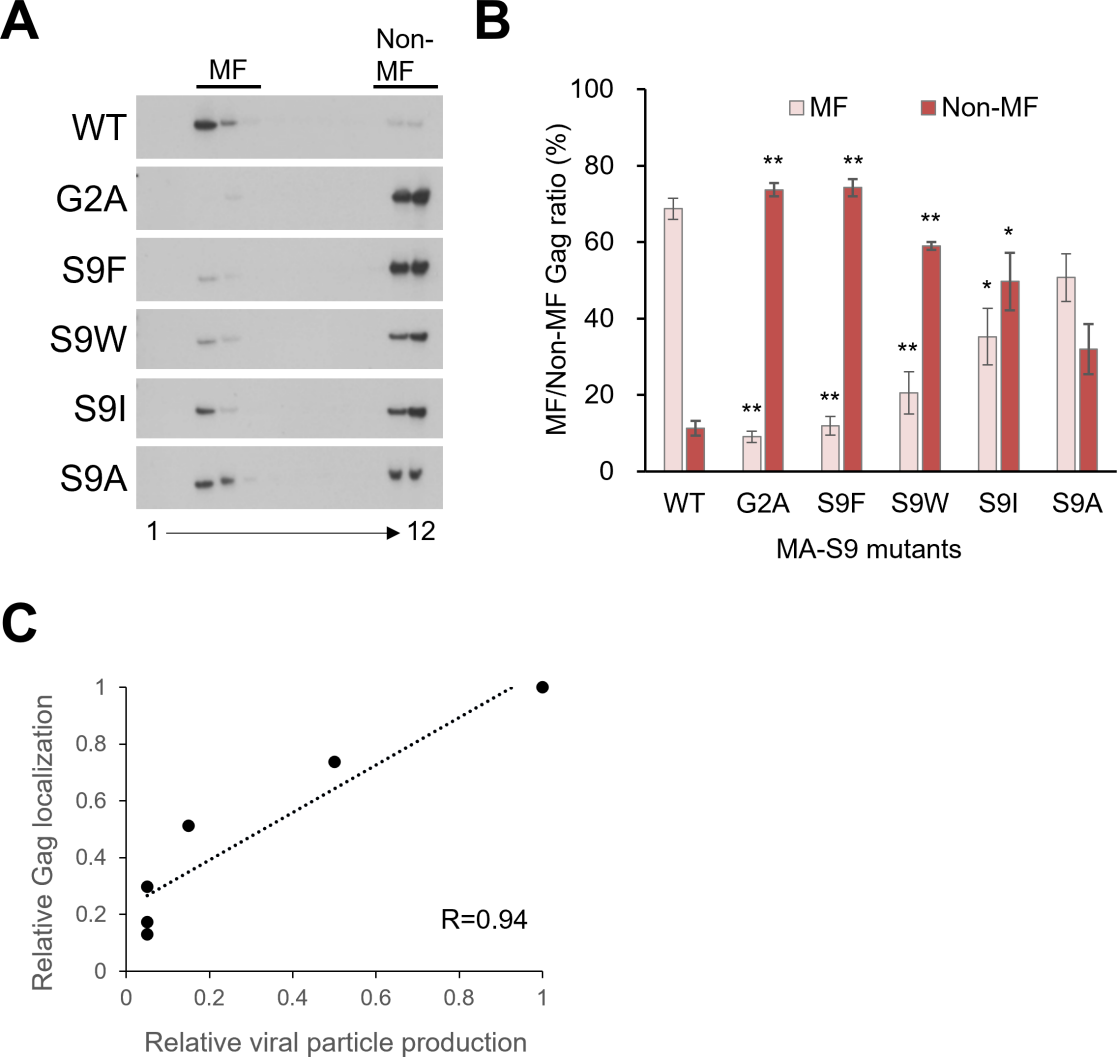
Effects of Gag-MA mutations on Gag localization to cell membranes. (**A**) HeLa cells were transfected with the indicated proviral mutants derived from pNL4-3ΔPro/ΔEnv as a parental WT clone. At 24 h post-transfection, cells were lysed to prepare the postnuclear supernatant for membrane flotation analysis (for details, see Materials and Methods). A sample in a sucrose solution (75%) was placed at the bottom of a centrifugation tube and layered with lowered concentrations of sucrose (65% and 10%) from bottom to top. After ultracentrifugation, fractions (1–12) were collected from centrifugation tubes (top to bottom) for examination of Gag by the Western blotting method. Gag proteins in the membrane fractions (MF; fraction numbers 3–5) and nonmembrane fractions (non-MF; fraction numbers 10 to 12) are indicated. Representative immunoblotting data from three independent experiments are shown. (**B**) The ratios of MF and non-MF Gag proteins for various clones are presented (mean ± SE). Significance relative to control NL4-3 was determined by Welch’s *t*-test. **P < 0.01; *P < 0.05. (**C**) The correlation between the relative gag localization and relative viral particle production was indicated by the *R*-score.

HIV-1 Gag proteins multimerize at PM during virion assembly (20, 56–58). To investigate Gag membrane targeting of Gag-MA mutants, which exhibit reduced capacities for virion production and Gag oligomerization, membrane flotation assays were performed (Fig. 9). Lysates of HeLa cells transfected with proviral ΔPro/ΔEnv clones were prepared and supplemented with high concentrations of sucrose and then overlaid with lower concentrations of sucrose in ultracentrifugation tubes. In this assay, membrane components float at the top of the sucrose layer in tubes after ultracentrifugation. Gag proteins fractionated from the top (membrane fraction: MF) to the bottom (non-membrane fraction: non-MF) portions were monitored by Western blotting analysis as previously described (15, 63). For the NL4-3 ΔPro/ΔEnv clone, Gag proteins were mainly detected in the MF (Fig. 9A and B). In contrast, Gag proteins of the control Gag-MA-G2A clone were marginally observed in the MF but predominantly observed in the non-MF. Among the Gag-MA-S9 mutants tested, the Gag-MA-S9I/A clones exhibited the membrane targeting of Gag proteins, albeit at levels lower than the corresponding activity in the WT. While a high proportion of Gag proteins in the MF was detected for the WT clone (MF/MF + non-MF ratio: ~70%), the Gag-MA-S9I and S9A mutants displayed MF/MF + non-MF ratios of about 40% and 50%, respectively. The results with the S9A mutant are consistent with those of the previous studies (22, 64). For the Gag-MA-S9F/W mutant clones, the levels of Gag proteins in the MF were very low relative to those in the non-MF. Of note, the Gag-MA-S9F clone showed a ratio of Gag proteins in the non-MF to total Gag proteins of around 80%, similar to the ratio for the Gag-MA-G2A clone. The Gag localization in the Gag-MA mutants tested seems to be related to their virion production (Fig. 9C; Pearson’s correlation coefficient, *R* = 0.94). Collectively, these results showed that the membrane targeting of Gag is significantly impaired by Gag-MA-S9 mutations, especially S9F/W, and the deficiency of Gag membrane targeting is well correlated with the remarkable reduction in virion production.

### Gag-MA-S9 mutations affect N-myristoylation of Gag proteins

**Fig 10:**
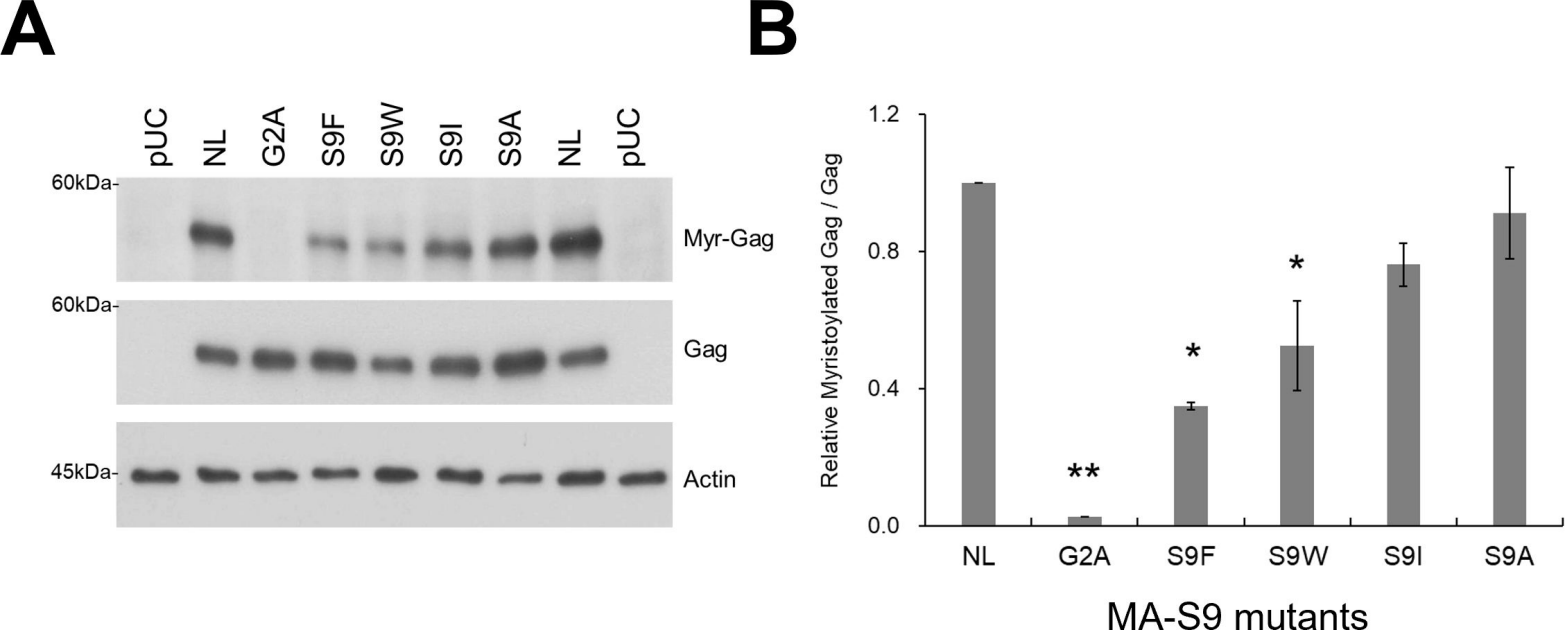
Effect of Gag-MA mutations on N-myristoylation of Gag proteins. (**A**) HEK293T cells were transfected with the indicated Gag-MA mutants derived from pNL4-3ΔPro/ΔEnv. At 24 h post-transfection, biotinylation reactions to detect any myristoylated proteins in cells were performed by using the Click-IT-based method (for details, see Materials and Methods). After the reactions, free biotin was removed from the samples, and equal amounts of samples (3.0 µg of total protein) were used for Western blotting analysis with horseradish peroxidase-conjugate streptavidin. Representative immunoblotting data from three independent experiments are shown. (**B**) Relative Myr-Gag/Gag is presented as described in Materials and Methods (mean ± SE). Significance relative to control NL4-3 was determined by Welch’s *t*-test. **P < 0.01; *P < 0.05. Myr-Gag, Myristoylated Gag; NL, NL4-3 WT; pUC, negative control.

Our Gag-MA mutants with deficiencies of virion production and Gag oligomerization showed a significant reduction in membrane-targeted Gag protein levels (Fig. 6A, 8 and 9). N-myristoylation at the G2 residue of Gag-MA is a prerequisite to PM targeting of Gag proteins (20, 56–58). It has been reported that N-terminal octapeptide amino acid sequences (G-X-X-X-S/T-X-X-X-, X, any amino acid) are important for the N-myristoylation reaction (65, 66). We thus examined whether Gag-MA-S9 mutations affect the N-myristoylation of Gag proteins (Fig. 10). Proviral ΔPro/ΔEnv clones were transfected into HEK293T cells, and any myristoylated proteins in the transfected cells were biotinylated through Click-iT reaction followed by Western blot analysis using streptavidin to visualize myristoylated proteins. As shown in Fig. 10, N-myristoylated Gag proteins were readily detected for NL4-3, whereas no N-myristoylated Gag proteins were observed for Gag-MA-G2A mutants. For Gag-MA-S9I/A mutant clones, N-myristoylated Gag proteins were modestly decreased compared to their levels in the WT. Interestingly, Gag-MA-S9F/W mutant clones produced barely detectable levels of N-myristoylated Gag proteins. These results showed that N-myristoylation of Gag is strongly inhibited by certain mutations at position 9 of Gag-MA. This reduction in myristoylated Gag proteins could explain the drastic reductions in Gag membrane targeting and subsequent virion production for the Gag-MA-S9F/W mutants. Together, our virological study showed that the hydrophobic amino acid substitutions at the MA-9 residue impair the elementary and overall processes of progeny virus particle formation in the cells.

## DISCUSSION

In this study, we revisited the role of the HIV-1 Gag N-terminal region from the viewpoint of its structure. Using *in silico* and experimental methods, we have obtained evidence that the HIV-1 Gag MA-9 residue is involved in the HIV-1 fitness *in vivo* and has the ability to modulate the level of disorder and motional dynamics of the Gag N-terminal peptide segment. Our study further demonstrated that the MA-9 residue is able to modulate the efficiency of progeny particle formation of HIV-1. Finally, we found that the level of virus particle formation is positively correlated with the levels of N-terminal disorder. To our knowledge, this is the first study showing the structural role of the MA-9 residue in parallel with the biological role of the Gag N-terminal disordered peptide segment.

Our study provides new insights into the regulation of the structure of the HIV-1 Gag N-terminal region. First, it provides evidence that a single hydrophobic amino acid substitution at the MA-9 site is sufficient to reduce the disorder potential of the MA N-terminal region (Fig. 4; Fig. S5). This finding is consistent with a principle of protein folding that intrinsically disordered segments of proteins comprise an insufficient proportion of hydrophobic amino acids to prevent folding (6, 50). Second, our NMR study revealed that a single hydrophobic substitution at the MA-9 site is sufficient to induce a reduction in motional dynamics of the MA N-terminal segment and conformational changes in the MA domain (Fig. 5). These findings disclose that the preservation of the non-hydrophobic residue at the MA-9 site is a prerequisite for maintaining high levels of structural disorder and dynamics of the Gag N-terminal segment.

Our study also provides new insights into the biological importance of the MA-9 residue. The MA-9 residue is placed immediately downstream of the N-myristoyl transferase recognition segment (G-X-X-X-S/T) (67) in the Gag MA N-terminal region (Fig. 3A). Previous studies have suggested that the MA-9 residue participates in biological processes such as plasma membrane targeting of the Gag precursor, virus release, and viral fusion with target cell membranes (22–24, 64, 68, 69). Our biological data (Fig. 6 to 10) are consistent with the previous findings, as well as with our structural data. Because molecular interactions are dependent on the structure and dynamics of the interaction regions, it is possible that changes in the disordered state and motional dynamics of the MA N-terminal region and MA conformation (Fig. 2, 4 and 5) influence early events during Gag translation, such as interaction of the Gag monomer with the N-myristoyl transferase. This, in turn, could impact the subsequent events involving the MA domain for viral particle formation, such as Gag protein binding to the tRNA for the Gag targeting to the plasma membrane (17–20), Gag–Gag interaction for the Gag oligomerization in the plasma membrane (30, 43), and incorporation of Env into the virions (21, 26, 31).

In addition, our comprehensive mutagenesis revealed an amino acid dependency of the mutational effects that was not disclosed in the previous studies; all of the single hydrophobic amino acid substitutions tested impair the HIV-1 particle formation, albeit to different degrees among the different mutations. In agreement with these findings, the hydrophobic amino acid at the MA-9 site was rarely detected in the public HIV-1 sequence database (Fig. 3B). These findings indicate that preservation of the non-hydrophobic residue at the MA-9 site is a prerequisite for optimizing the viral particle formation in the cells and the fitness of HIV-1 *in vivo*.

Finally, our study provides a novel insight into the significance of the retention of the unstructured state of the Gag N-terminal region. We found that the level of viral particle production is positively correlated with the level of the disorder potential of the Gag N-terminal segment (Fig. 6B). This implies that the preservation of a high level of disorder in the Gag N-terminal segment is critical for the optimal production of virus particles. Such physiological significance of the disordered region of a protein terminus has also been reported for human UDP-α-D-glucose-6-dehydrogenase, in which the C-terminal disordered segment plays a key role in controlling the structural dynamics of the enzyme to favor inhibitor binding (54).

In summary, the present findings clarify the relation between microscopic and macroscopic aspects of the HIV-1 survival strategy. Our data indicate that the Gag MA-9 residue plays a key role in maintaining the unstructured state and motional dynamics of the Gag N-terminal segment in order to optimize progeny virus production in host cells. This model provides a rational explanation for the continued disorder of the N-terminal component of the HIV-1 Gag precursor protein across the evolutionary history of HIV-1.

## MATERIALS AND METHODS

### Prediction of the disordered segment of the N-terminal region of HIV-1 MA

Disorder scores of the first 30 amino acid residues of the N-terminus of the MA domain were estimated with AIUPred (51) and PONDR VL-XT predictor (33–35) using the Gag full-length and MA domain sequence from the strain HIV-1 NL4-3 (GenBank accession no. AF324493) (36).

### Molecular modeling of the HIV-1 Gag precursor

A three-dimensional model of the Gag monomer of the HIV-1 NL4-3 strain (GenBank accession no. AF324493) (36) was constructed using the reported structures of the HIV-1 Gag subdomains. Briefly, individual structures of Gag subdomains, such as MA, CA, NC, and p6, were constructed by homology modeling and connected with the overlapped regions by using the Molecular Operating Environment (MOE; Chemical Computing Group, Montreal, Quebec, Canada). The structures of the Gag subdomain were obtained from the Protein Data Bank (the PDB IDs were MA: 2H3I [46]; MA-CA: 1L6N [70]; CA-SP1: 5I4T [71]; CA-SP1-NC: 1U57 [72]; NC: 1A1T [73]; and p6: 2C55 [74]). The structure of SP2, which is composed of 14 amino acids, is unavailable at present; we constructed SP2 using the Protein builder application of MOE. The obtained full-length Gag precursor model was optimized via energy minimization using the AMBER10:Extended Hückel Theory (EHT) force field in MOE, which combines the Amber10- and EHT-bonded parameters for large-scale energy minimization (75). The model was subjected to MD simulations. Briefly, the simulations were performed using the pmemd.cuda.MPI module in the Amber 16 program (32) with the ff14SB force field for protein simulation (76). The Gag precursor model was solvated in a truncated box of TIP3P water molecules with a distance of at least 9 Å around the model (77). The Gag precursor model was neutralized and ionized in 150 mM NaCl using the tleap program of Amber software. The size of the system with Gag precursor, water molecules, and ion molecules was 179.11 Å height, 123.13 Å width, and 138.46 Å length (Fig. S6). A non-bonded cut off of 10 Å was used. Bond lengths involving hydrogen were constrained with SHAKE, a constraint algorithm that satisfied Newtonian motion (78). The trajectory data of all MD simulations were collected at 2 fs intervals. After heating calculations were performed for 20 ps up to 310K using the NVT ensemble, simulations were executed using the NPT ensemble at 310K under 1 atm in 150 mM NaCl for a total of 200 ns. The trajectory files during MD simulations were used to calculate the RMSD. RMSDs between the heavy atoms of the initial complex structure and the structure at given time points during the MD simulation were calculated to monitor the overall structural changes. The molecular surface of each Gag region was calculated using the Linear Combinations of Pairwise Overlaps algorithm (79). Calculations of the RMSDs and molecular surface were done by the *cpptraj* module in AmberTools 16, a trajectory analysis tool (32).

### Molecular modeling of the HIV-1 Gag dimer

A Gag dimer model of HIV-1 was constructed by the MOE using the Gag monomer obtained at 200 ns of MD. The CA CTD dimer model (PDB ID: 4COP) (45) was used as a template for the Gag dimer interface. First, two Gag models were placed around the CTD–CTD interface on an axis and separated until there was no atomic clash. Next, the two Gag models without Gag–Gag interaction were subjected to MD simulations under the same calculation conditions as described above. The size of the system with Gag precursor, water molecules, and ion molecules was 145.16 Å height, 112.65 Å width, and 85.00 Å length (Fig. S6). The simulations were executed for a total of 1,000 ns. The trajectory files during MD simulations were used to calculate the number of hydrogen bonds between two Gag precursors. The formation of hydrogen bonds was determined using geometric criteria, i.e., the distance and angle between an acceptor heavy atom and a donor heavy atom. Calculations of RMSDs and the number of hydrogen bonds were done by the *cpptraj* module in AmberTools 16, a trajectory analysis tool (32).

### Analysis of the molecular surface area

Trajectory files during MD simulations were used to calculate the molecular surface area of the Gag precursor subdomains (MA, CA, SP1, NC, SP2, and p6) and functional regions of viral particle production (MA-HBR, MA-H2H3 loop, MA-H5H6 loop, CA-MHR, CA-SP1 junction, CA-NTD NTD interaction sites, Zn-zinc finger, and p6-PTAP) using the linear combinations of pairwise overlaps algorithm (79) in *cpptraj* operated in AmberTools 16 (32).

### Analysis of the Gag length

The trajectory files during MD simulations were used to calculate distances of N-terminal-end-to-C-terminal-end of the Gag monomer and Gag subdomains (MA, MA-CA, MA-CA-SP1, MA-CA-SP1-NC, and MA-CA-SP1-NC-SP2) using the distance application in *cpptraj* operated in AmberTools 16 (32).

### Molecular patch analysis

The interaction-prone areas on the Gag precursors were estimated using the Protein Patch Analyzer tool in MOE. A dimer model of the Gag after 1,000 ns of MD simulation was used for the patch analyses. The MA domain model was obtained from the PDB (PDB ID: 2H3F) (46). Briefly, the Protein Patch Analyzer tool was applied to search for the hydrophobic patches (minimal patch area of 50 Å^2^) that were potentially involved in the interactions with the hydrophobic moieties of molecules.

### Shannon entropy analysis

The amino acid variation at each position of Gag-MA was analyzed with Shannon entropy as described previously (15, 80). Full-length matrix amino acid sequences of HIV-1 (*n* = 25,222) were obtained from the HIV Sequence Database (https://www.hiv.lanl.gov/content/index). Shannon entropy was calculated on the basis of Shannon’s equation (49):


$$H\left(i\right)=-\sum_{x_{i}}p\left(x_{i}\right)\log_{2}p\left(x_{i}\right)\;\;\;\;\;\;\;\;\;\;\;\;\;\;\;\;\;\;\;\;\;\;\left(x_{i}=G,A,I,V,\ldots \right),$$

where *H(i*), *p(x_i_*), and *i* indicate the amino acid entropy score for an individual position, the probability of occurrence of a given amino acid at that position, and the number of the position, respectively. An *H(i)* score of zero indicates absolute conservation, whereas a score of 4.4 bits indicates complete randomness.

### Plasmid construction and protein preparation for NMR analysis

The DNA sequence encoding Gag MA (residues 1–132) was codon-optimized, synthesized, and inserted into pET21a by VectorBuilder Inc to obtain the protein with a C-terminal 6× histidine-tag (6His), referred to as Gag-MA 6His. An S9F mutant, Gag-MA-S9F 6His, was constructed using inverse PCR. The resulting constructs were confirmed by DNA sequencing.

Gag-MA 6His was expressed in *Escherichia coli* BL21(DE3)/pLysS cells. Cells were grown in M9 minimal medium containing 1 g/L ^15^NH_4_Cl as the sole nitrogen source for the preparation of uniformly ^15^N-labeled protein. Protein purification was performed at 4°C using a Ni Sepharose High Performance column (Cytiva) and HiLoad 16/60 Superdex 200 column (GE Healthcare). The obtained protein was dissolved in 25 mM sodium phosphate, pH 5.5, 200 mM NaCl, and 1 mM 1,4-dithiothreitol (DTT). Gag-MA-S9F 6His was also prepared in the same manner. The resulting protein solutions were concentrated using an Amicon Ultra-15 3 kDa (Merck Millipore). Sample concentrations were determined by measuring UV absorbance at 280 nm, utilizing a molar extinction coefficient of 16,960.

### NMR experiments

All NMR spectra were acquired at 308 K using a Bruker AVANCE III HD 600 MHz spectrometer (Bruker, Billerica, MA) equipped with a cryogenic probe. NMR data processing was performed using TopSpin 3.5.7. An NMR sample containing 100 µM ^15^N-labeled Gag-MA 6His, 25 mM sodium phosphate, pH 5.5, 200 mM NaCl, 1 mM DTT, and 5% (vol/vol) D_2_O was used to record a 2D ^1^H–^15^N HSQC spectrum. NaOH titration was conducted on the NMR sample, and 2D ^1^H–^15^N HSQC spectra were acquired under five different pH conditions (pH 6.0, 6.5, 7.0, 7.5, and 8.0) for signal assignment. Subsequently, a 2D ^1^H–^15^N HSQC spectrum of 100 µM ^15^N-labeled Gag-MA-S9F 6His was measured in 25 mM sodium phosphate, pH 5.5, 200 mM NaCl, 1 mM DTT, and 5% (vol/vol) D_2_O. The steady-state {^1^H}-^15^N NOE was measured by recording two interleaved spectra: one with a ^1^H presaturation time of 3 s and a relaxation delay of 2 s for the NOE experiment, and the other with a 5 s relaxation delay for the reference experiment. In this experiment, NMR samples containing 300 µM of the respective proteins at pH 5.5 were employed. Proton–nitrogen heteronuclear NOE values were calculated as the ratio between the cross-peak intensities with (I) and without ($I_{0}$) ^1^H presaturation ($I/I_{0}$). The errors were estimated from the root mean square of the baseline noise in the two spectra (81).

### Plasmid DNAs

The full-length proviral clone pNL4-3, the Pro- and Env-deficient proviral clones pNL4-3ΔPro/ΔEnv (15), HaloTag-fused Gag (62), and NanoLuc-fused Gag (82) have been described previously (15, 62, 82). Their site-specific Gag-MA and Gag-CA mutants were generated by the standard PCR-based mutagenesis method.

### Cells

Monolayer cell lines HEK293T (ATCC CRL-1573), HeLa (ATCC CCL-2), and HeLa-derived reporter TZM-bl (83) were cultured and maintained in Eagle’s minimal essential medium containing 10% heat-inactivated fetal bovine serum.

### Assays for virion production and single-cycle infectivity

HEK293T cells were transfected with a full-length pNL4-3 or its mutant clones by Lipofectamine 2000 (Invitrogen), and the culture supernatants were collected at 24 h post-transfection. Virion-associated reverse transcriptase (RT) activity in the culture supernatants was measured by RT assays as previously described (84, 85) and used as an index of the virion production level. Equal amounts of viruses (10,000 RT units) were inoculated into TZM-bl cells (4 × 10^3^). On day 2 post-infection, cells were lysed and subjected to luciferase assays (Promega) to assess viral single-cycle infectivity.

### Analysis of Env incorporation into virions

Proviral clones were transfected into HEK293T cells by the calcium phosphate coprecipitation method. On day 2 post-transfection, the supernatant was passed through a 0.45 µm filter to remove cell debris, layered on top of 25% sucrose solution, and ultracentrifuged at 25,000 rpm for 2 h at 4°C (Himac CP80NX, P40ST rotor; Eppendorf Himac Technologies, Ibaraki, Japan). After centrifugation, the pellets were dissolved in 1× TNE buffer, and then the amounts of virions were monitored by using an HIV-1 p24 antigen enzyme-linked immunosorbent assay kit (ZeptoMetrix Corporation, Buffalo, NY). The samples were analyzed by the Western blotting method using anti-HIV-1 Gag-p24 (183-H12-5C; NIH AIDS Reagent Program; catalog no. 3537), anti-HIV1 gp120 (ab21179, abcam), and anti-β-actin clone AC-15 (Sigma-Aldrich Co.) antibodies as described previously (86). Signal intensities of Gag proteins detected by immunoblotting were quantitated with Fusion Edge Software (Vilber Lourmat).

### Analysis of Gag oligomerization by crosslinking

The crosslinking experiments to assess Gag oligomerization were performed as previously described (60) with some modifications. HeLa cells were transfected with pNL4-3ΔPro/ΔEnv or its derivatives by Lipofectamine 2000, and at 24 h post-transfection, cells were washed with PBS and then treated with 0.1 mM EGS (ethylene glycol bis [succinimidyl succinate]; FUJIFILM Wako Pure Chemical Corporation) or PBS for 30 min at room temperature. Cells were then treated with 0.1 M Tris-HCl, pH 7.5, to quench the EGS reaction, lysed, and subjected to Western blot analysis as described above. Immunoblotting analysis was performed using anti-HIV-1 p55 + p24 + p17 (ab63917; abcam) and anti-β-actin clone AC-15 (Sigma-Aldrich Co.) antibodies.

### NanoBRET assays

Assays were performed as described previously (15). Briefly, vectors encoding HaloTag-fused Gag and NanoLuc-fused Gag were cotransfected into HEK293T cells. On day 2 post-transfection, NanoBRET activity was measured by the NanoBRET Nano-Glo detection system (Promega).

### Membrane flotation assays

HeLa cells were transfected with pNL4-3ΔPro/ΔEnv or its derivatives. At 24 h post-transfection, cells were treated with hypotonic lysis buffer (10 mM Tris-acetate, pH 7.4) for 15 min at 4°C. Cells were then homogenized by 30–40 strokes in a Downs homogenizer. A one-ninth volume of 10× salt buffer (10 mM Tris-acetate, pH 7.4, 500 mM KCl, and 1,000 mM NaCl) was added, and the solution was mixed. After centrifugation at 300 g for 10 min at 4°C, the post-nuclear supernatants were adjusted to 75% sucrose in 1× salt buffer by mixing with 87% sucrose, and 1.5 mL of the mixture was added to a centrifugation tube (13 PA tube; Eppendorf Himac Technologies). On top of the mixture, 6.9 mL of 65% sucrose and 2.4 mL of 10% sucrose were layered, and the tubes were ultracentrifuged at 98,400 × *g* for 20 h at 4°C using a Himac CP80NX and P40ST rotor (Eppendorf Himac Technologies). After ultracentrifugation, 12 fractions were collected from the top to the bottom and stored at −20°C until analysis. Fractionated samples were examined for Gag proteins by Western blotting as described above. Signal intensities of Gag proteins detected by immunoblotting were quantitated with Fusion Edge Software (Vilber Lourmat).

### Analysis of the N-myristoylation of Gag proteins

HEK293T cells were transfected with pNL4-3ΔPro/ΔEnv or its derivatives by Lipofectamine 2000 (Invitrogen). The culture medium was changed at 6–8 h post-transfection, and Click-iT Myristic Acid Azide (12-Azidododecanoic Acid; Thermo Fisher Scientific) was added to the culture medium (final concentration 25 µM). At 24 h post-transfection, cells were washed with PBS, lysed in myr-buffer (50 mM Tris-HCl, pH 8.0, 1% SDS, protease inhibitor cocktail [Sigma], and 250 U/mL Benzonase [Sigma]), and incubated for 20 min at 4°C. After vortexing for 5 min, supernatants were collected by centrifugation at 18,000 × *g* for 5 min at 4°C. The preparations thus obtained (100 µg of total protein for each) were reacted with biotin-alkyne (final concentration 40 µM) using a Click-iT Protein Reaction Buffer Kit (Thermo Fisher Scientific) according to the manufacturer’s instructions, and free biotin was removed using Zeba Dye and Biotin Removal Spin Columns (Thermo Fisher Scientific). The resulting samples were subjected to Western blot analysis using streptavidin–horseradish peroxidase conjugate (Thermo Fisher Scientific) to detect any biotinylated proteins. The immunoblot was reacted with anti-HIV-1 p55 + p24 + p17 (ab63917; abcam) to confirm the migration position corresponding to Gag proteins. Signal intensities of Gag proteins detected by immunoblotting were quantitated with Fusion Edge Software (Vilber Lourmat). After obtaining the signal intensities of myristoylated Gag (Myr-Gag), total Gag, and β-actin of each sample, those of Myr-Gag and total Gag were normalized by that of β-actin. The myristoylation ratio of Gag was calculated as Myr-Gag/Gag signal intensities in each sample relative to that in NL4-3.

### Statistics and reproducibility

Differences between mutant groups were evaluated by Welch’s *t*-test. Error bars indicated the SDs of three independent experiments. The *R* values of the relation among the disorder level, viral production, infectivity, Env incorporation, and Gag localization were calculated by Pearson’s correlation coefficient. The number of experimental replicates is indicated in the legends for Fig. 6A, 7B, 8B, 9B, and 10B and in the corresponding Materials and Methods section.

## Data Availability

All data needed to evaluate the conclusions in the paper are present in the paper and supplemental material.

## SUPPLEMENTAL MATERIAL

Figures S1 to S6. jvi.00887-25-s0001.pdf Analysis data on the structural characteristics of HIV-1 Gag.

ASM does not own the copyrights to Supplemental Material that may be linked to, or accessed through, an article. The authors have granted ASM a non-exclusive, world-wide license to publish the Supplemental Material files. Please contact the corresponding author directly for reuse.
