# Supplementary material for: Positive correlation between structural disorder of the HIV-1 Gag N-terminal segment and progeny virus particle formation
Source: J Virol. 2025 Sep 16;99(10):e00887-25. doi: 10.1128/jvi.00887-25 (PMC12548442; doi:10.1128/jvi.00887-25)
Supplement: Figures S1 to S6. — Analysis data on the structural characteristics of HIV-1 Gag. [file jvi.00887-25-s0001.pdf]

Supplementary Figures

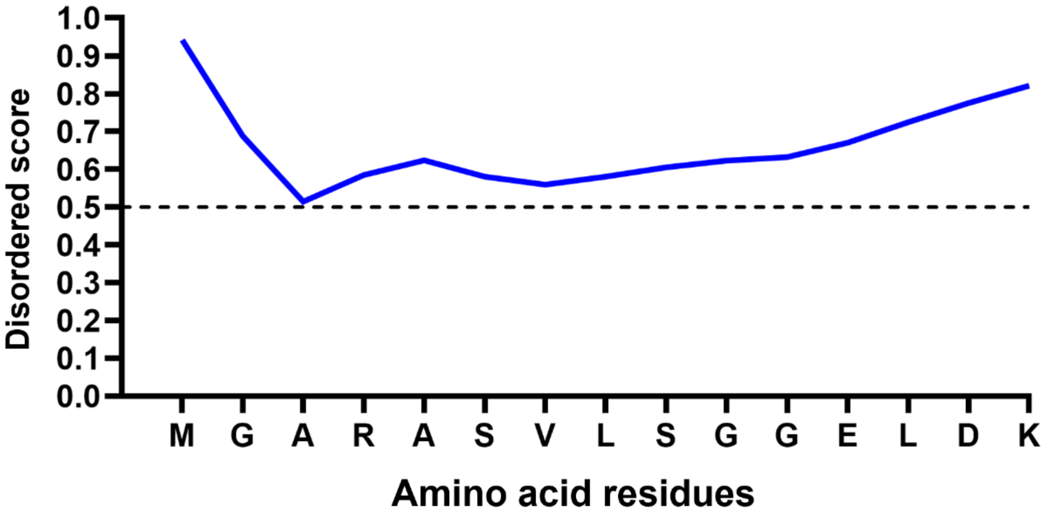

Supplementary Figure 1: Disorder scores of N-terminal regions of Gag protein. Disorder scores of first 15 residues of the Gag N-terminal region in Figure 1A are indicated.

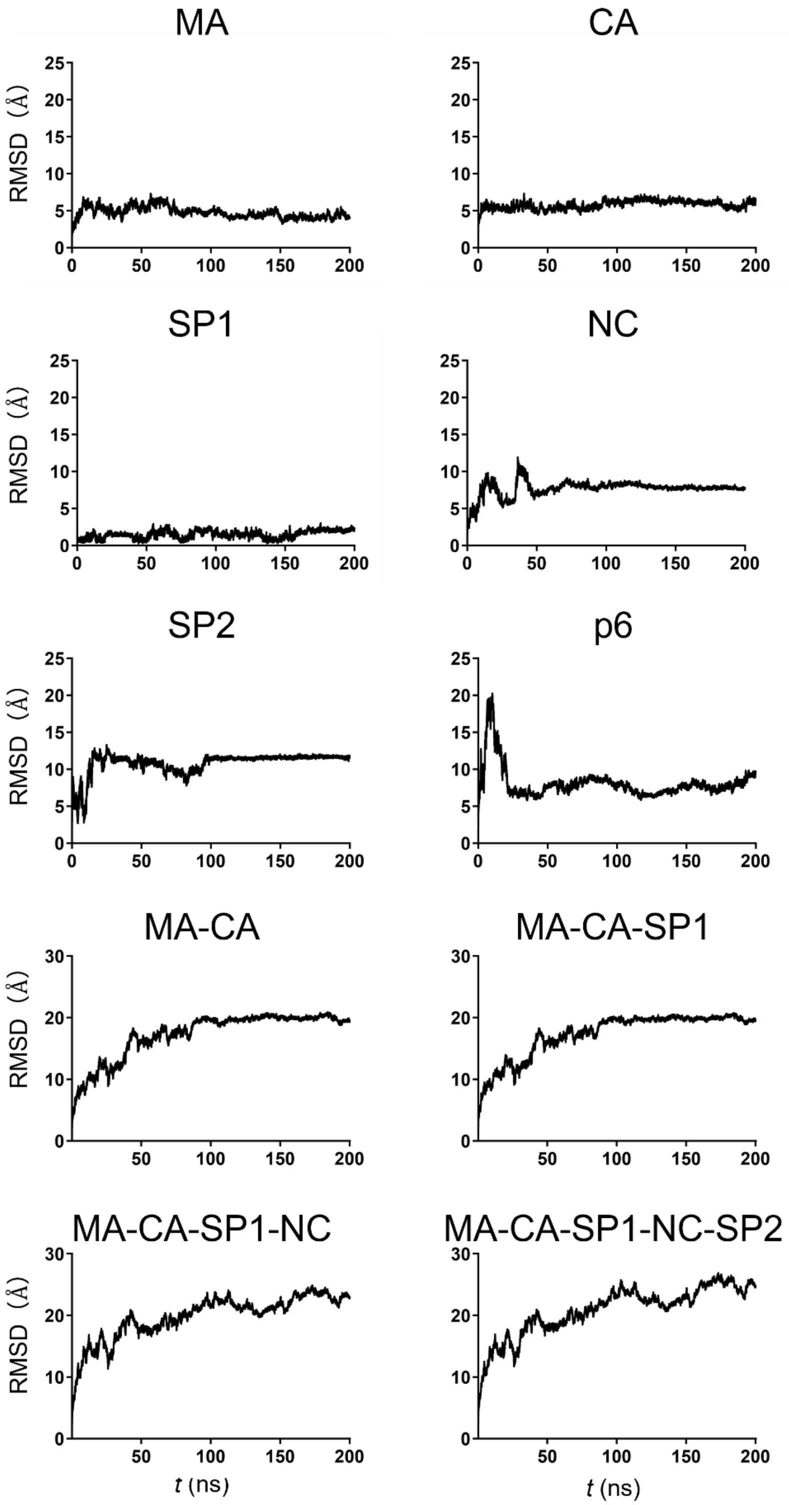

Supplementary Figure 2: Structural changes of Gag subdomains and multi-domains connected by unstructured linkers during MD simulations. The RMSDs of the Gag subdomains during 0 to 200 ns

of MD simulations were calculated using the cpptraj module in AmberTools 16 as described previously (1, 2).

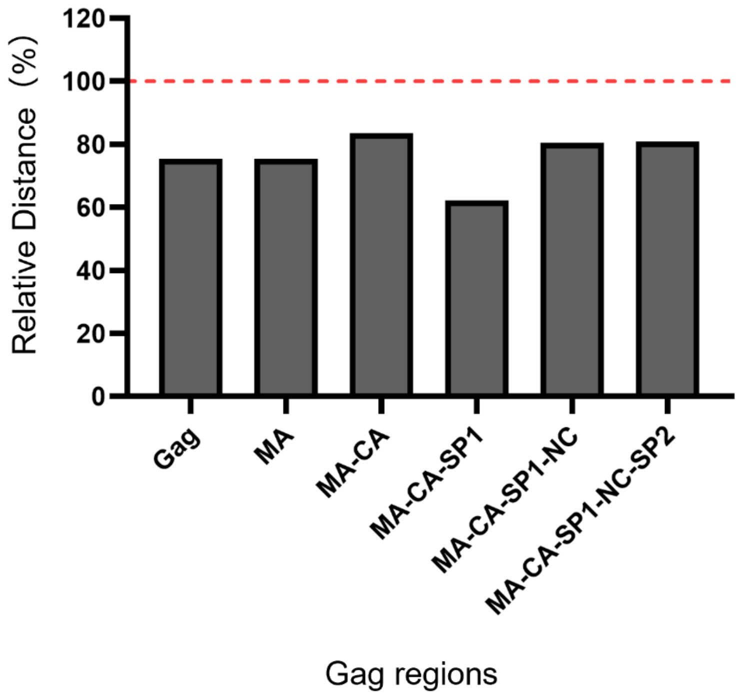

Supplementary Figure 3: Analysis of the Gag compaction in solution. End-to-end distances of indicated Gag regions were calculated using the distance application in *cpptraj* operated in AmberTools 16 (3). Relative distances after MD simulations as compared with those before MD simulations are indicated.

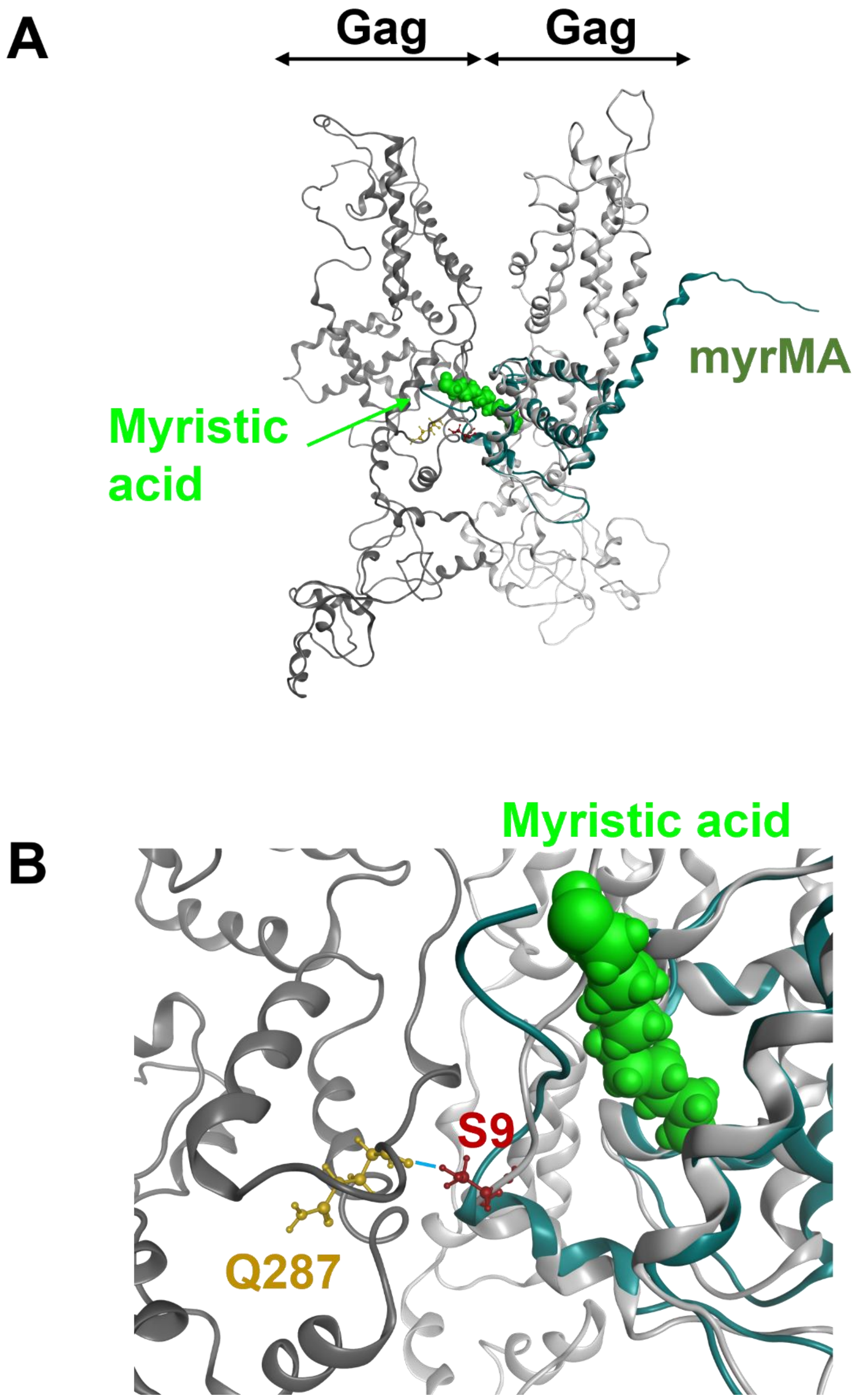

Supplementary Figure 4: Superposition between the Gag dimer and myrMA. (A) The MD-based Gag dimer model (Figure 2E) was superimposed on the reported myristate sequestered MA structure (PDB ID:

2H3I) (4) using the Structure Superposition tool in MOE. (B) Enlarged view around a hydrogen bond between the S9 and Q287 residues in the Gag dimer model. Greenish spheres indicate the myristic acid at glycine 2 of the Gag MA N-terminus. The blue bar between residues S9 and Q287 indicates the hydrogen bond.

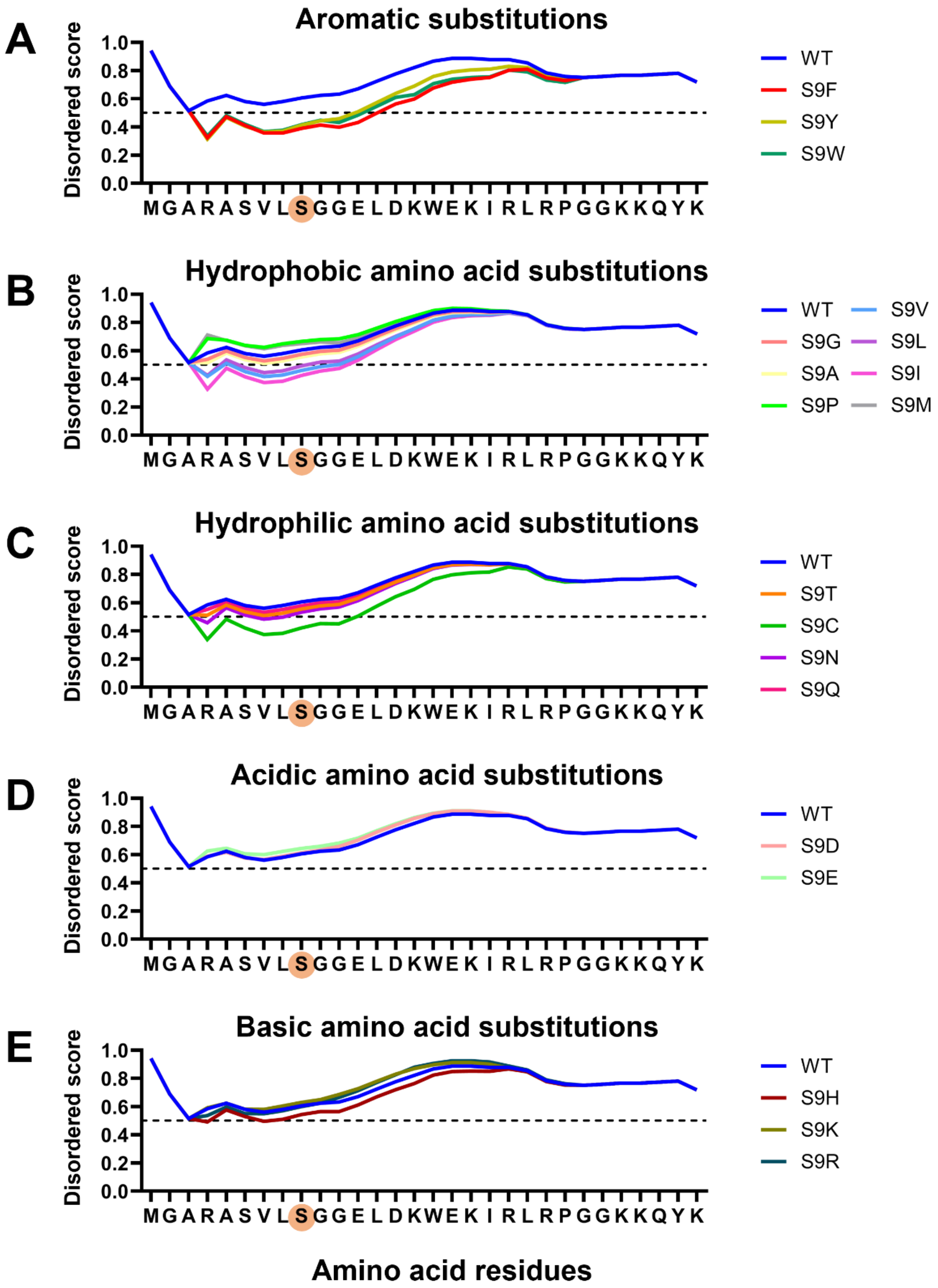

19 **Supplementary Figure 5: Prediction of the disordered segment of the MA N-terminal region using**

PONDR. Disorder scores of the first 30 amino acid residues of the N-terminus of the MA domain were estimated with PONDR VL-XT predictor (5-7) using the sequence from the HIV-1 NL4-3 strain (GenBank accession no. AF324493) (8). The dashed lines at the Y axis of the figures are the threshold lines for disordered/structured residues.

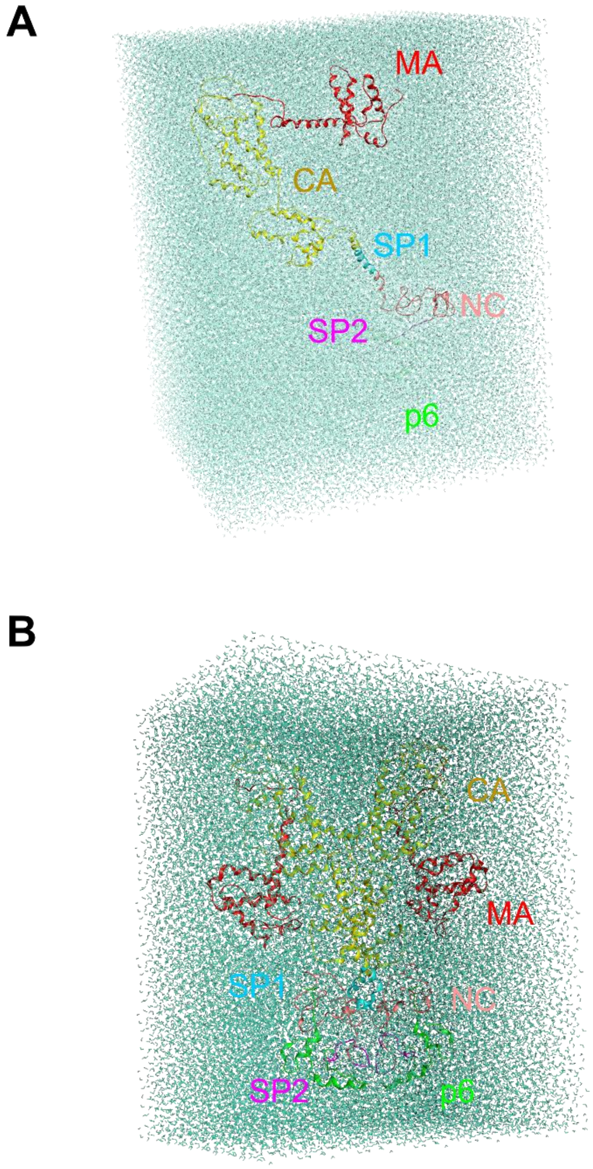

Supplementary Figure 6: The scale of MD simulation system. The MD simulations were performed using the pmemd.cuda.MPI module in the Amber 16 program (3) with the ff14SB force field for protein simulation (9). The Gag monomer (A) or dimer (B) models were solvated in truncated box of TIP3P water molecules with a distance of at least 9 Å around the model (10).

Supplementary References

1. Izumida M, Kotani O, Hayashi H, Smith C, Fukuda T, Suga K, Iwao M, Ishibashi F, Sato H, Kubo Y. 2022. Unique Mode of Antiviral Action of a Marine Alkaloid against Ebola Virus and SARS-CoV-2. Viruses 14.
2. Matsuda M, Hirai-Yuki A, Kotani O, Kataoka M, Zheng X, Yamane D, Yokoyama M, Ishii K, Muramatsu M, Suzuki R. 2024. Loxapine inhibits replication of hepatitis A virus in vitro and in vivo by targeting viral protein 2C. PLoS Pathog 20:e1012091.
3. D.A. Case RMB, D.S. Cerutti, T.E. Cheatham, III, T.A. Darden, R.E. Duke, T.J. Giese, H. Gohlke, A.W. Goetz, N. Homeyer, S. Izadi, P. Janowski, J. Kaus, A. Kovalenko, T.S. Lee, S. LeGrand, P. Li, C. Lin, T. Luchko, R. Luo, B. Madej, D. Mermelstein, K.M. Merz, G. Monard, H. Nguyen, H.T. Nguyen, I. Omelyan, A. Onufriev, D.R. Roe, A. Roitberg, C. Sagui, C.L. Simmerling, W.M. Botello-Smith, J. Swails, R.C. Walker, J. Wang, R.M. Wolf, X. Wu, L. Xiao and P.A. Kollman. 2016. AMBER 16. University of California, San Francisco.
4. Saad JS, Miller J, Tai J, Kim A, Ghanam RH, Summers MF. 2006. Structural basis for targeting HIV-1 Gag proteins to the plasma membrane for virus assembly. Proc Natl Acad Sci U S A 103:11364-9.
5. Romero P, Obradovic Z, Li X, Garner EC, Brown CJ, Dunker AK. 2001. Sequence complexity of disordered protein. Proteins 42:38-48.
6. Li X, Romero P, Rani M, Dunker AK, Obradovic Z. 1999. Predicting Protein Disorder for N-, C-, and Internal Regions. Genome Inform Ser Workshop Genome Inform 10:30-40.
7. Romero, Obradovic, Dunker K. 1997. Sequence Data Analysis for Long Disordered Regions Prediction in the Calcineurin Family. Genome Inform Ser Workshop Genome Inform 8:110-124.
8. Adachi A, Gendelman HE, Koenig S, Folks T, Willey R, Rabson A, Martin MA. 1986. Production of acquired immunodeficiency syndrome-associated retrovirus in human and nonhuman cells transfected with an infectious molecular clone. J Virol 59:284-91.
9. Maier JA, Martinez C, Kasavajhala K, Wickstrom L, Hauser KE, Simmerling C. 2015. ff14SB: Improving the Accuracy of Protein Side Chain and Backbone Parameters from ff99SB. J Chem Theory Comput 11:3696-713.
10. William L. Jorgensen, Jayaraman Chandrasekhar, Jeffry D. Madura, Roger W. Impey, Michael L. Klein. 1983. Comparison of simple potential functions for simulating liquid water. The Journal of Chemical Physics 79:926-935.
